# TRPA1 channels promote astrocytic Ca^2+^ hyperactivity and synaptic dysfunction mediated by oligomeric forms of amyloid-β peptide

**DOI:** 10.1186/s13024-017-0194-8

**Published:** 2017-07-06

**Authors:** Anthony Bosson, Adrien Paumier, Sylvie Boisseau, Muriel Jacquier-Sarlin, Alain Buisson, Mireille Albrieux

**Affiliations:** 1 University Grenoble Alpes, Grenoble Institut des Neurosciences, GIN, Chemin Fortuné Ferrini, BP170, F-38000 Grenoble, France; 2 Inserm, U1216, F-38000 Grenoble, France

**Keywords:** Astrocyte, Calcium, Alzheimer, Synapse, TRPA1, APP/PS1

## Abstract

**Background:**

Excessive synaptic loss is thought to be one of the earliest events in Alzheimer’s disease (AD). However, the key mechanisms that maintain plasticity of synapses during adulthood or initiate synapse dysfunction in AD remain unknown. Recent studies suggest that astrocytes contribute to functional changes observed during synaptic plasticity and play a major role in synaptic dysfunction but astrocytes behavior and involvement in early phases of AD remained largely undefined.

**Methods:**

We measure astrocytic calcium activity in mouse CA1 hippocampus *stratum radiatum* in both the global astrocytic population and at a single cell level, focusing in the highly compartmentalized astrocytic arbor. Concurrently, we measure excitatory post-synaptic currents in nearby pyramidal neurons.

**Results:**

We find that application of soluble Aβ oligomers (Aβo) induced fast and widespread calcium hyperactivity in the astrocytic population and in the microdomains of the astrocyte arbor. We show that astrocyte hyperactivity is independent of neuronal activity and is repaired by transient receptor potential A1 (TRPA1) channels blockade. In return, this TRPA1 channels-dependent hyperactivity influences neighboring CA1 neurons triggering an increase in glutamatergic spontaneous activity. Interestingly, in an AD mouse model (APP/PS1–21 mouse), astrocyte calcium hyperactivity equally takes place at the beginning of Aβ production, depends on TRPA1 channels and is linked to CA1 neurons hyperactivity.

**Conclusions:**

Our experiments demonstrate that astrocytes contribute to early Aβo toxicity exhibiting a global and local Ca^2+^ hyperactivity that involves TRPA1 channels and is related to neuronal hyperactivity. Together, our data suggest that astrocyte is a frontline target of Aβo and highlight a novel mechanism for the understanding of early synaptic dysregulation induced by soluble Aβo species.

**Electronic supplementary material:**

The online version of this article (doi:10.1186/s13024-017-0194-8) contains supplementary material, which is available to authorized users.

## Background

Formation of toxic amyloid-β (Aβ) species and their accumulation in plaques are key hallmarks in the pathogenesis of Alzheimer’s disease (AD) but it has now been recognized that, far ahead of plaques formation, soluble Aβ oligomers (Aβo) are the pathology-triggering species [1]. More specifically, Aβo manage a progressive loss of synaptic connectivity leading to neurodegeneration. Astrocytes are important safeguards of synaptic function and it becomes increasingly evident that these cells accomplish a more important role in brain function than previously thought. The loss of synapses may reflect functional downfall of astrocytes. These cells possess receptors and signaling machinery for all known neurotransmitters thus sensing neuronal activity [2]. They also actively secrete gliotransmitters such as ATP, glutamate, D-serine hence modulating activity of neuronal receptors [3, 4]. Consequently, through their involvement in the tripartite synapse, they both sense and modulate synaptic output [5].

Unlike neurons, astrocytes are electrically non-excitable cells but they are equipped with numerous channels, receptors or exchangers triggering Ca^2+^ signals. Thus, astrocytic excitability is based on highly spatiotemporally coordinated fluctuations of intracellular Ca^2+^ concentration relying on plasmalemmal and intracellular channels [6]. Important progress has been made in studying astrocytes branches Ca^2+^ signaling since they are the primary sites for interactions with neurons [7–9]. Direct monitoring of Ca^2+^ dynamics in the processes of adult mouse hippocampal astrocytes has revealed intense local and compartmentalized activity that is dissociated from activity in the cell body [8]. However, until recently, it was difficult to specifically explore this astrocytic calcium activity since channels and receptors involved in astrocytic calcium signaling were commonly expressed in neurons. However, among the receptors involved in astrocytic calcium signaling, transient receptor potential A1 (TRPA1) channel seems to be specifically expressed in astrocytes and absent from neurons within hippocampus *stratum radiatum* [10, 11]. The discovery of TRPA1 channel as an important mediator of Ca^2+^ entry restricted to astrocytes in the mouse hippocampus provided new opportunity to explore astrocyte signaling in relation to neuron-astrocyte interaction particularly in case of synaptic dysregulation.

In late transgenic AD mouse models, i.e. in phases associated with plaques formation, it has been shown that astrocytic calcium activity dramatically increases, becomes synchronous nearby Aβ plaques and coordinates calcium signals at long distance [12]. Within somatosensory cortex, this astrocyte hyperactivity around plaques is mediated by purinergic signaling [13]. Surprisingly, astrocytes behavior and reactions in early phases of AD remained largely undefined despite their probable involvement in the progressive loss of synaptic connectivity and in the complex and critical cellular phase of AD [14]. We thus chose to study the impact of soluble Aβ oligomers on astrocytic function at the onset of early defensive cellular phase well before astrogliosis or inflammatory processes.

In this work, we monitored astrocytic calcium activity within the CA1 *stratum radiatum* region of the mouse hippocampus both at the astrocytic population level and at a single cell level, focusing in the astrocytic arbor. We characterized spontaneous Ca^2+^ signaling properties at these two related levels and showed that Aβo exposition induced at once a global and a local Ca^2+^ hyperactivity. We showed that this hyperactivity was independent of neuronal activity and was totally restored to physiological level by blocking TRPA1 channels. This TRPA1 channels-dependent influence of Aβo on astrocyte activity consequently impacted neighboring CA1 neurons, increasing glutamatergic spontaneous activity. In an AD mouse model, we showed that astrocyte hyperactivity was an early phenomenon setting up at the onset of Aβ production, being also related to neuronal hyperactivity and preceding TRPA1 channel overexpression. Overall, our data provide a novel mechanism for the understanding of early toxicity of soluble Aβo species.

## Methods

### Slice preparation

Coronal hippocampal slices (ranging from 300 to 350 μm thick) were prepared from Swiss mice (postnatal day 17–23; Janvier, Le Genest St Isle, France) or APP/PS1–21 transgenic mice [15] (postnatal day 19–28) together with control littermates. Mice were killed by decerebration and decapitated. The brain was rapidly removed and cut in ice-cold cutting ACSF containing (in mM): 2.5 KCl, 7 MgCl_2_, 0.5 CaCl_2_, 1.2 NaH_2_PO_4_, 25 NaHCO_3_, 11 D-glucose and 234 sucrose bubbled with 95% O_2_ and 5% CO_2_, with a vibratome VT1200S (Leica, Wetzlar, Germany). Slices containing the hippocampus were placed in ACSF containing (in mM): 126 NaCl, 2.5 KCl, 1.2 MgCl_2_, 2.5 CaCl_2_, 1.2 NaH_2_PO_4_ bubbled with 95% O_2_ and 5% CO_2_ and supplemented with 1 mM sodium pyruvate at room temperature for a recovery period.

### Slices bulk loading with Fluo-4 AM.

Briefly, 350 μm coronal slices were loaded with the calcium indicator dye Fluo-4 by immersion for 45 min at 35 °C in a bath containing 5 μM Fluo-4 AM (Life Technologies), 0.01% Pluronic acid F-127 (Molecular Probes), 0.005% Cremophor EL (Sigma-Aldrich) and 0.05% DMSO (dimethyl sulfoxide, Sigma-Aldrich) in ACSF. The loading chamber was continuously oxygenated with 95% O_2_ and 5% CO_2_. Slices were then transferred into dye-free ACSF for at least 45 min prior to experiments. Mainly live astrocyte took up the fluorescent dye with these conditions [16, 17].

### Single-astrocyte dye loading with Fluo-4

Coronal 300 μm slices were transferred to a chamber allowing constant perfusion with ACSF at room temperature, bubbled with 95% O_2_ and 5% CO_2_ on the stage of an upright compound microscope (Eclipse E600 FN, Nikon, Paris, France) equipped with a water immersion 60× objective (NA 1.0) and an infrared-differential interference contrast optics with CCD camera (Optronis VX45, Kehl, Germany). Glass pipettes 8–11 MΩ (Harvard apparatus) were filled with intracellular solution containing (in mM): 105 K-gluconate, 30 KCl, 10 phosphocreatine, 10 HEPES, 4 ATP-Mg, 0.3 GTP-Tris, 0.2 Fluo-4 pentapotassium salt (Life Technologies), adjusted to pH 7.2 with KOH. Signals were amplified by Axopatch 200B, sampled by a Digidata 1440A interface and recorded with pClamp8 software (Molecular Devices, Foster City, USA). Astrocytes were identified based on morphological, localization in the *stratum radiatum* and negative resting potential (between −70 and −80 mV). Input resistance was calculated by measuring current in response to a 10 mV pulse with 80 ms duration, near the end of the voltage command. Only passive astrocytes showing linear *I/V* relationship and low input resistance (~ 50 MΩ) were kept for dye loading. After achieving whole-cell configuration, access resistance was constantly monitored, and astrocytes were excluded from this study when this parameter varied >20% throughout the experiment. To allow sufficient diffusion of the dye and avoid astrocyte dialysis, the time in whole-cell configuration was limited to less than 5 min. Then, the patch pipette was carefully withdrawn to allow the astrocyte to recover. In order to maximize the diffusion of the dye into the astrocytic processes, we waited at least 15 min before calcium imaging [8, 18].

### Calcium imaging

Bulk or single-astrocyte loaded slices were placed in a constantly perfused chamber on the stage of an upright compound microscope (Eclipse E600 FN, Nikon, Paris, France) equipped with a water immersion 40× (NA 0.8) or 60× (NA 1.0) objective and a confocal head (confocal C1 head, Nikon, Paris, France). Excitation was achieved with light at 488 nm and emission was filtered with a 515 ± 15 nm filter. Images were acquired with EZ-C1 software (Nikon, Paris, France) at 1.2 s intervals in a single confocal plane over a period of 5 min.

### Bulk loading calcium imaging data analysis

Prior to analysis, raw images were stabilized (when needed if slight *x-y* drift occurred during recordings, z drifts were excluded) using ImageJ plugin Template Matching. *CalSignal* software was used to measured intracellular Ca^2+^ activity, analyzing the fluorescence signal F within each region of interest (ROI) corresponding to the cell body area of each astrocyte [19]. F_0_ was calculated for each ROI on the recording period. Based on the ΔF/F_0_ ratios, significant fluorescence variations were detected and a Ca^2+^ event was defined as a significant and continuous signal increase larger than a fixed threshold followed by a significant and continuous signal decrease larger than the same threshold. Thus, cells were defined as active when fluorescence increased ≥2 standard deviations relative to baseline fluorescence. After peak detection, each Ca^2+^ transients were visually checked by the operator. The theoretical Poisson distribution was calculated by the method of least squares approximating λ until it most closely fits the observed frequency distribution.

### Single astrocyte loading data analysis

Ca^2+^ transients were measured in two-dimensional images, in individual subregions matching the shape of the astrocyte structure. Manually selected ROIs (~1 μm^2^) were placed along astrocytic processes lying in the focal plane [8] and a ROI was also selected in the soma if accessible. Prior to analysis, raw images were stabilized (when needed if slight *x-y* drift occurred during recordings, z drifts were excluded) using ImageJ plugins Template Matching and filtered with 3D Hybrid Median Filter [7]. *CalSignal* software was used to measure intracellular Ca^2+^ activity, analyzing the fluorescence signal F within each ROI. As described above, significant changes in fluorescence were detected on the basis of the calculated ΔF/F_0_ ratios. Each Ca^2+^ transients within ROI were visually checked by the operator and reported in a raster plot in order to discriminate focal activities from expanded ones (as described by [8]). At the end of each recording, z-stacks (0.5 μm steps) were performed to obtain tri-dimensional projections of astrocyte territories revealed by Fluo-4 loading. Images were then filtered with 3D Hybrid Median Filter plugin in ImageJ.

### Electrophysiological recordings

Whole-cell recordings were made from the somata of visually identified CA1 pyramidal neurons. Patch pipettes (4–6 MΩ) were filled with an internal solution containing (in mM): 105 K-gluconate, 30 KCl, 10 phosphocreatine, 10 HEPES, 4 ATP-Mg, 0.3 GTP-Tris, 0.3 EGTA, adjusted to pH 7.2 with KOH. Spontaneous excitatory post-synaptic currents (sEPSCs) were collected at a membrane holding potential of −65 mV which is close to the reverse potential of GABA. All recordings were done at room temperature (22–24 °C) and only a single neuron was studied per slice. sEPSCs and their kinetics were analyzed in 5-min epochs within the time frame of the recordings. Each epoch was compared to the initial 5-min recording and sEPSCs frequencies were normalized to this initial value. Access resistance was constantly monitored and recordings were excluded from this study when this parameter varied >20% throughout the experiment. Recordings were analyzed using the Clampfit module of the pClamp8 software (Molecular Devices, Foster City, USA) with a threshold at −20 pA to exclude miniature EPSCs.

### Aβ oligomerization, monomer purification and drug application

Recombinant Aβ_1–42_ peptide (Bachem) was resuspended in 1,1,1,3,3,3-hexafluoro-2-propanol (HFIP) to 1 mM until complete resuspension as described previously [20]. Following HFIP evaporation, Aβ oligomers were prepared by diluting Aβ to 1 mM in DMSO, then to 100 μM in ice-cold ACSF with immediate vortexing and bath sonication, and then incubated at 4 °C for 24 h with mild agitation.

When appropriate, the Aβ monomer is purified on a C18 column (SPE-Chromabond-HRX C18 ec, 200 μl, 5 mg, Macherey-Nagel, France). The column was equilibrated with 0.1% trifluoroacetic acid (TFA) in water. Immediately after dilution in DMSO, the Aβ sample was loaded and the column was washed three times with 0.1% TFA. Then, a gradient of acetonitrile from 30 to 60% was applied (Additional file 1). Fractions (0.1 ml) were collected. The elution profile was determined by measuring the absorbance at 275 nm. The peak fraction was collected and the concentration of peptide was determined by absorbance at 275 nm using ɛ_275 nm_ = 1400 M^−1^ cm^−1^. The peptide is then stored at −80 °C.

Aβo, Aβm, tetrodotoxin (TTX; Latoxan, Valence, France), Ca^2+^-free solution (ACSF - 0 Ca^2+^ - 1 mM EGTA) and HC 030031 (Sigma-Aldrich) were bath applied at the appropriate concentration during 5 min before and during calcium imaging or electrophysiological recordings. Minocycline hydrochloride (Sigma-Aldrich) was bath applied during 15 min before and during calcium imaging recording.

### Immunohistochemistry

Mice were deeply anesthetized with 10% chloral hydrate and perfused intracardially with 10 ml 0.9% NaCl followed by 35 ml 4% paraformaldehyde in 0.1 M PBS, pH 7.3. Brains were rapidly removed, post-fixed overnight at 4 °C in 4% paraformaldehyde, immersed in 20% sucrose in 0.1 M PBS, pH 7.5 overnight, frozen in cooled (−35 °C) isopentane and stored at −30 °C. Serial frontal sections (30 μm thick) were cut with a cryostat microtome (HM 500 M, Microm, Francheville, France). Sections were blocked by incubation with 3% bovine serum albumin in TBS-Tween-Triton (TBSTT) (0.1 M Tris Base, 0.15 M NaCl, 0.1% Tween, 0.1% Triton X-100) for 30 min (dilution/blocking buffer). Tissue sections were then incubated overnight at 4 °C with either an anti-NeuN antibody (AbCys, France, mouse monoclonal; 1:500), anti-GFAP antibody (Molecular Probes, USA, mouse monoclonal; 1:1000), anti-TRPA1 antibody (Novus, USA, rabbit polyclonal; 1:100) or anti-Iba-1 antibody (Wako, USA, rabbit polyclonal; 1:500). Tissue sections were washed in TBSTT and incubated for 2 h at room temperature with Cyanin 3- (Jackson Immuno Research Laboratories, USA; 1:1000) or Alexa 488-conjugated secondary antibodies (Life Tecchnology, USA; 1:1000). Sections were washed in TBSTT and mounted in Dako fluorescent mounting medium (Dako, USA).

When appropriate, amyloid-β deposits were stained using Thioflavine S [21]. Sections were re-hydrated in TBS buffer (0.1 M Tris Base, 0.15 M NaCl), incubated in filtered 1% aqueous Thioflavine S (Sigma, France) for 8 min at room temperature, in the dark and washed several times in TBS buffer.

### Image acquisition

Sections were examined with a Zeiss LSM 710 confocal laser scanning microscope with a Plan Apochromat 20× objective (NA 0.8) or an oil immersion Plan Neofluor 40× objective (NA 1.3) and translating platform with motorized crossed roller stages. When appropriate, mosaics were acquired for each channel separately with “Zen” software, in a 12-bit format, using the tile scan function. For TRPA1 and GFAP co-staining, sections were also acquired with a Zeiss Airyscan module with an oil immersion Plan Apochromat 63× objective (NA 1.46) to improve lateral resolution (~140 nm) and signal-to-noise ratios. For illustration, images were merged with ImageJ software.

### Immunoblotting

Dissected hippocampi from 1-month old APP/PS1–21 mice were homogenized in cold buffer containing 0.32 M sucrose and 10 mM HEPES, pH 7.4. Samples were maintained at 4 °C during all steps of the experiments. Homogenates were cleared at 1000 x g for 10 min to remove nuclei and large debris. Samples in loading buffer were boiled for 10 min and equal amounts of proteins (20 μg, quantified by micro-BCA assay (Pierce) in duplicate extracts) were resolved on a 4–20% gradient Bis-Tris polyacrylamide precast stain free gels (Bio-Rad) in denaturing conditions. Proteins were transferred to a polyvinylidene difluoride membrane (Millipore) for 30 min at 4 °C. Membranes were blocked with 3% dry milk in Tris-Buffered Saline (TBS: 10 mM Tris, 150 mM NaCl, pH 7.4) containing 0.1% Tween for 1 h at room temperature. Membranes were probed with anti-TRPA1 antibody (Novus, USA; 1:2000) and anti-GFAP antibody (Dako, USA, rabbit polyclonal; 1:100000) diluted in 3% dry milk in 0.1% Tween TBS overnight at 4 °C. Membranes were washed in 0.2% Tween TBS and probed with HRP-conjugated anti-rabbit IgG (Fab’) (Interchim, France; 1:40,000) antibody for 45 min at room temperature. After washes, specific proteins were visualized with an enhanced chemiluminescence ECL Detection System (Bio-Rad) and the chemidoc system (Bio-Rad). Chemiluminescence signals were normalized to protein loading signals acquired using Stain-free pre-cast gels (Bio-Rad).

### Statistical analysis

Data were analyzed using R (the R Project for Statistical Computing) [22]. Comparisons between two groups were conducted with the two-tailed Mann-Whitney test. Kruskal-Wallis test followed by Pairwise comparison using Wilcoxon rank sum test was used when needed for multiple comparisons. Proportions of hyperactive/active astrocyte and focal/expanded activities were compared with χ^2^-test. Data were expressed as mean ± SEM accompanied by distribution of experimental points. Graphic significance levels were *, *p* < 0.05; **, *p* < 0.01 and ***, *p* < 0.001.

## Results

### Ca^2+^ activity in the astrocytic population and individual processes in mouse hippocampus

Ca^2+^ signals encoding is known to be different in astrocytic cell body versus processes and involves different calcium sources such as internal stores release or external entry. Spatio-temporal Ca^2+^ activity characteristics within astrocyte correlate to specific function associated with different territories. Astrocytes not only operate as individual cells but also take part in functional network through gap-junction coupling allowing remote communication in delimited functional brain area. We studied astrocytic Ca^2+^ activity at once in the global astrocytic population and in single cell microdomains on mouse hippocampal slices (P17-P23) by using two complementary imaging techniques: i) Fluo-4 AM bulk loading to record calcium activity in the astrocytic population and ii) whole-cell patch-clamp technique to load individual astrocytes with Fluo-4 dye giving access to single cell processes territory (Additional file 2).

**Fig. 1:**
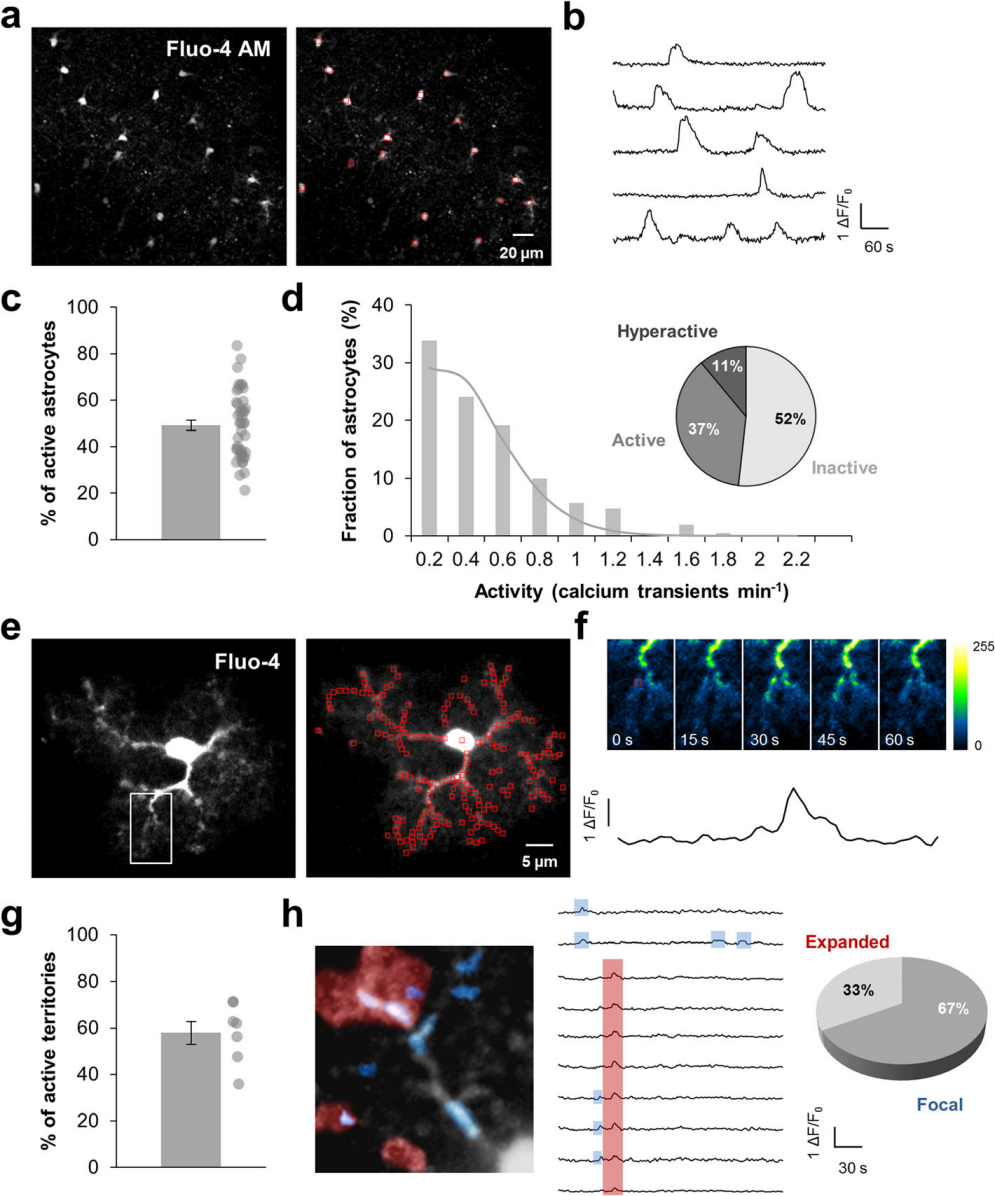
Detection of global and compartmentalized Ca^2+^ events in astrocytes. **a** Fluo-4-loaded astrocytes in the *stratum radiatum* of a mouse coronal slice. Fluorescence variations were analyzed in astrocyte cell bodies (*red square*). **b** Example of typical fluorescence variations recorded in physiological condition. **c** Proportion of astrocytes displaying calcium activity during a 5-min recording. Each dot corresponds to individual value for each recorded slice (*n* = 43). **d** Frequency histogram showing the occurrence of Ca^2+^ events in the astrocyte population and the related theoretical Poisson distribution (*grey curve*). Pie chart representing inactive (0 event per min), active (0.2–0.6 event per min) and hyperactive (> 0.6 event per min) astrocytes in physiological condition. **e** Single patch-clamp Fluo-4-loaded astrocyte in the *stratum radiatum* of a mouse coronal slice. Fluorescence variations within processes were analyzed in subregions of ~1 μm^2^ (*red square*) in a single z-plane. **f** Time-lapse Ca^2+^ imaging in an astrocyte process showing an example of Ca^2+^ event. Time between frames, 15 s. The *black curve* corresponded to the fluorescence signal recorded in a selected ROI (*red square*, first image). **g** Proportion of subregions displaying a calcium activity during a 5-min recording. Each dot corresponds to individual value for each recorded astrocyte (*n* = 7). **h** Artificial color superposition of focal Ca^2+^ events (1–4 contiguous subregions, *blue*) and expanded Ca^2+^ events (> 4 contiguous subregions, *red*) occurring during a 5-min recording in an astrocyte process, as defined by [8]. Traces showing occurrence of Ca^2+^ peaks in contiguous subregions with blue and red highlights identifying focal (*blue*) and expanded (*red*) Ca^2+^ events respectively. In physiological condition, focal events represent the major part of Ca^2+^ events within the astrocytic processes

We analyzed signals by positioning regions of interest (ROIs) either in each cell body of Fluo-4 AM loaded astrocytes (20.0 ± 1.3 ROIs by frame; *n* = 43 slices from 14 animals in physiological condition; Fig. 1a, b) or by subdividing the entire patch-clamp loaded territory into subregions of similar area (1 μm^2^; 111.7 ± 7.3 ROIs by astrocyte; *n* = 7 astrocytes from 6 animals in physiological condition; Fig. 1e, f). These subregions corresponded to functional microdomains as previously defined by Di Castro et al. [8] since their size matched approximately the synaptic density in the neuropil region. We investigated some of the temporal properties of astrocytic Ca^2+^ signals (such as the proportion of active cells, the proportion of active microdomains, the frequency of events) and some of the spatial properties of Ca^2+^ signals within the astrocytic arbor (such as the events propagation).

**Fig. 2:**
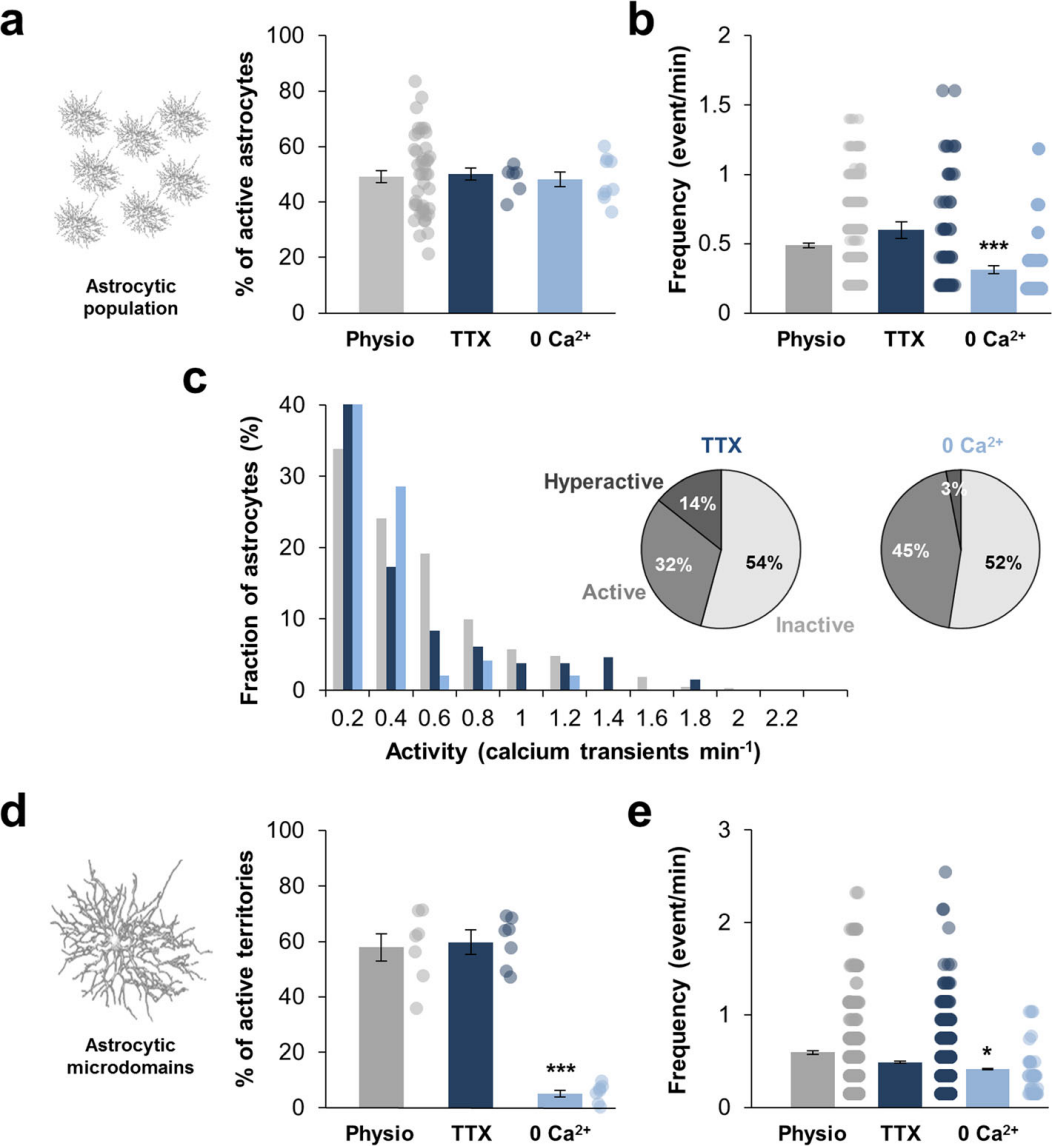
Spontaneous astrocytic Ca^2+^ events are fully autonomous and partly depend on external Ca^2+^ entry. **a**, **b** Within the astrocytic population, proportion of astrocytes displaying calcium activity and frequency of astrocyte calcium activity in physiological condition (*grey*; *n* = 43), under 500 nM TTX application (*dark blue*; *n* = 6) or in Ca^2+^-free medium (0 Ca; *light blue*; *n* = 9). Each dot corresponds to individual value for each recorded slice (proportion) or each recorded ROI (frequency). **c** Frequency histogram revealing that 500 nM TTX had no effect on the frequency distribution whereas significantly less astrocytes were hyperactive in Ca^2+^-free medium (0 Ca^2+^; *light blue*). **d**, **e** Within single astrocyte arbor, proportion of subregions displaying calcium activity and frequency of astrocyte calcium activity in physiological condition (*grey*; *n* = 7), under 500 nM TTX application (*dark blue*; *n* = 7) or in Ca^2+^-free medium (0 Ca^2+^; *light blue*; *n* = 7). Each dot corresponds to individual value for each recorded astrocyte (proportion) or each recorded ROI (frequency). Results are compared with the physiological condition with *, *p* < 0.05; **, *p* < 0.01 and ***, *p* < 0.001


In physiological condition, 49.1 ± 2.2% of bulk loaded astrocytes were spontaneously active during a 5 min recording period (*n* = 43 slices from 14 animals) with a mean frequency of 0.49 ± 0.01 event/min (*n* = 817 astrocytes; Fig. 1c). Frequency histograms revealed that the occurrence of Ca^2+^ events in the astrocyte population was similar to a Poisson distribution with λ = 1.85 suggesting that spontaneous Ca^2+^ event within *stratum radiatum* was a stochastic phenomenon (Fig. 1d). Based on an in vivo study performed in mouse cortical astrocytes [13], we classified astrocytes as inactive (0 event/min), active (0.2–0.6 event/min) and hyperactive (> 0.6 event/min). We observed a minority of hyperactive astrocytes (11%) within mouse hippocampus *stratum radiatum* in physiological condition (Fig. 1d). This spontaneous activity is independent of neuronal activity since TTX (500 nM) had no effect on either the proportion of active astrocytes or the frequency of calcium activity or the proportion of active/hyperactive astrocyte (Fig. 2a–c). This therefore confirmed that the global activity of the astrocytic population is totally autonomous within the mouse hippocampus *stratum radiatum* [18]. In single patch-clamp loaded astrocyte, we had access to Ca^2+^ activity in a two-dimensional acquisition plane representing on average 1945.3 ± 140.1 μm^2^ (*n* = 7 astrocytes; Fig. 1e). We analyzed spatio-temporal encoding of Ca^2+^ signals in 111.7 ± 7.3 microdomains within the astrocytic arbor. In physiological condition, 57.9 ± 4.8% of these microdomains were active (i.e. at least 1 Ca^2+^ event; Fig. 1f, g) during a 5 min recording period with a mean activity frequency of 0.59 ± 0.01 event/min (*n* = 782 ROIs). As previously described in mouse dentate gyrus astrocytes [8], we identified two types of Ca^2+^ events: “focal events” that are confined to 1 to 4 microdomains and “expanded events” appearing in more than 4 contiguous subregions (Fig. 1h). An additional movie file shows these two types of events in more details (Additional file 3). Focal events were the large majority (67%) occurring randomly in all subregions. Expanded events arose less often (33%) and spread over 7.1 ± 0.4 μm^2^. Within *stratum radiatum*, these two types of Ca^2+^ event were independent of neuronal activity since TTX application didn’t affect either the proportion or the frequency of focal and expanded events (Fig. 2d, e).

Additional file 3:Expanded and focal Ca^2+^ events in astrocytic processes. Time-lapse imaging of calcium activity in Fluo-4 loaded astrocytic processes in a 5-min recording. Focal and expanded events were pseudo-colored in blue and red respectively (left panel). Raw data were shown in the right panel. (MP4 10286 kb)


Astrocytic Ca^2+^ signaling involves both internal Ca^2+^ stores and external Ca^2+^ entry. Within the *stratum radiatum* astrocyte population, removal of external Ca^2+^ from the bath (ACSF - 0 Ca^2+^ - EGTA 1 mM) had no effect on the proportion of spontaneously active astrocytes (48.2 ± 2.7% in Ca^2+^-free medium vs 49.1 ± 2.2% in ACSF; *n* = 9 slices from 5 animals; *p* = 0.97) but aborted approximately 35% of events within active cells (0.31 ± 0.02 event/min in Ca^2+^-free medium vs 0.48 ± 0.01 in physiological condition; *n* = 103 astrocytes; *p* < 0.0001) meaning that one third of somatic Ca^2+^ events depended on a Ca^2+^ entry (Fig. 2a–c). Particularly, the proportion of hyperactive/active astrocytes was strongly affected (3% of hyperactive astrocytes versus 11% in physiological condition; *p* < 0.001). Yet, location of internal stores is not uniformly distributed in the astrocytic territory since they are concentrated in cell body and thick processes, thin astrocytic processes being practically devoid of Ca^2+^ stores [23]. Consistently, we observed that removing external Ca^2+^ suppressed nearly all the compartmentalized Ca^2+^ transients in the astrocytic arbor (5.1 ± 1.3% active territories vs 57.8 ± 4.8% in physiological condition; *n* = 7 astrocytes from 6 animals; *p* = 0.0006) and reduced the frequency of the residual signals (0.41 ± 0.01 event/min vs 0.59 ± 0.01 in physiological condition; *n* = 687 ROIs; *p* = 0.04) (Fig. 2d, e).

### Astrocytes become hyperactive in presence of amyloid-β oligomers

**Fig. 3:**
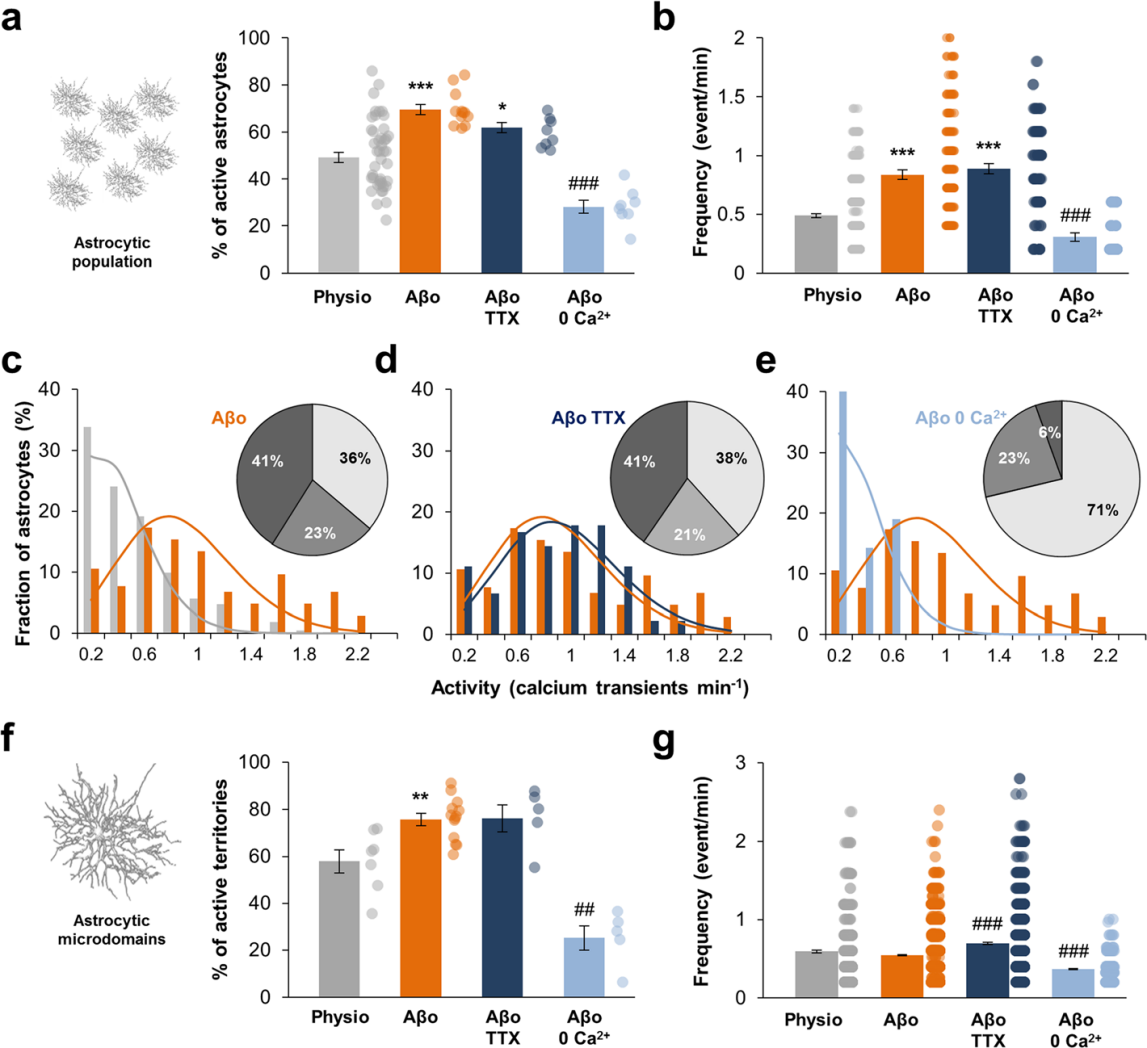
Extracellular Aβo application induces astrocytes hyperactivity. **a**, **b**. Within the global astrocytic population, proportion of astrocytes displaying calcium activity and frequency of astrocyte calcium activity in physiological condition (*grey*; *n* = 43), under 100 nM Aβo application (*orange*; *n* = 12), 100 nM Aβo + 500 nM TTX co-application (*dark blue*; *n* = 8) and 100 nM Aβo in Ca^2+^-free medium application (0 Ca^2+^; *light blue*; *n* = 8). **c**, **d**, **e**. Frequency histogram revealing that significantly more astrocytes were hyperactive and less were inactive under 100 nM Aβo application or 100 nM Aβo + 500 nM TTX co-application. Aβo-induced hyperactivity is abolished in Ca^2+^-free medium. Related theoretical Poisson distributions were shown accordingly. **f**, **g**. Within single astrocyte arbor, proportion of subregions displaying calcium activity and frequency of astrocyte calcium activity in physiological condition (*grey*; *n* = 7), under 100 nM Aβo application (*orange*; *n* = 13), 100 nM Aβo + 500 nM TTX co-application (*dark blue*; *n* = 5) and 100 nM Aβo in Ca^2+^-free medium application (0 Ca^2+^; *light blue*; *n* = 5). Results are compared with the physiological condition with *, *p* < 0.05; **, *p* < 0.01 and ***, *p* < 0.001 or the Aβo condition with #, *p* < 0.05; ##, *p* < 0.01 and ###, *p* < 0.001

To investigate the effect of soluble Aβ_1–42_ oligomers (Aβo) on astrocytic calcium signaling, we perfused hippocampal slices with 100 nM of oligomeric forms of the peptide during 5 min before recording. In the global astrocytic population, application of Aβo resulted in a significant increase in the proportion of active astrocytes (69.7 ± 2.1% vs 49.1 ± 2.2% in physiological condition; *n* = 12 slices; *p* < 0.0001; Fig. 3a). Within active cells, the frequency of Ca^2+^ events also significantly rose (0.83 ± 0.04 event/min vs 0.48 ± 0.01 event/min in physiological condition; *n* = 237 astrocytes; *p* < 0.0001; Fig. 3b). Frequency histogram was switched to high frequency and developed differently from the basal Poisson distribution (λ = 4.39; Fig. 3c). This suggested that Ca^2+^ event was not anymore a stochastic phenomenon but was managed by Aβo. The fraction of hyperactive astrocyte (i.e. > 0.6 event/min) was significantly larger (41% in Aβo condition vs 11% in physiological condition; *p* = 0.012; Fig. 3c). Thus, under application of 100 nM Aβo, there is an increase in the population of hyperactive astrocytes at the expense of basal-active and -inactive astrocytes.

In individual patch-clamped astrocyte, application of Aβo induced a significant increase of the fraction of active territories (75.8 ± 2.5% vs 57.9 ± 4.8% in physiological condition; *n* = 13 astrocytes; *p* = 0.0055; Fig. 3f) with no effect on the frequency of Ca^2+^ events within these microdomains (0.54 ± 0.01 vs 0.59 ± 0.02 event/min; *n* = 1806 ROIs; *p* = 0.178; Fig. 3g). Both focal and expanded event numbers were increased and the ratio of expanded/focal Ca^2+^ events was not significantly affected by Aβo (41% of expanded events in Aβo condition vs 33% in physiological condition; *p* = 0.27; Additional file 4a). However, expanded events size significantly increased compared to physiological condition (9.2 ± 0.5 μm^2^ vs 7.1 ± 0.4 μm^2^; *p* = 0.045; Additional file 4c).

Application of monomeric forms of Aβ_1–42_ (Aβm) had no effect on either the proportion of active cells or the Ca^2+^ signal properties within cells confirming that only oligomeric forms of Aβ were responsible for this Ca^2+^ hyperactivity (*n* = 14 slices from 4 animals; Additional file 1c). Major oligomers present in our samples were low-molecular weight oligomers (2- to 4- mers; Additional file 1a).

Thus, 100 nM Aβo triggered astrocytic calcium hyperactivity within the *stratum radiatum* as soon as 5 min in the global astrocytic population, manifested as an increase of the proportion of active cells along with an increase of the frequency of individual Ca^2+^ events. Interestingly, this global hyperactivity went along with a sustained increase of Ca^2+^ activity within astrocytic processes including an increase of the proportion of active microdomains together with an increase of the expanded events size in the astrocytic processes.

### Aβo-induced astrocytic Ca^2+^ hyperactivity is independent of neuronal activity or microglia activation and involves external Ca^2+^ entry

Aβo could potentially affect directly and/or indirectly Ca^2+^ signaling within astrocytes. In order to ascertain the neuronal part in the astrocyte hyperactivity, we co-applied 500 nM TTX with 100 nM Aβo. In bulk loading condition, TTX had no effect on the Aβ-induced astrocytic Ca^2+^ hyperactivity either in the proportion of active astrocytes (61.9 ± 2.2%; *n* = 8 slices from 6 animals; Fig. 3a) or in the frequency of Ca^2+^ events (0.89 ± 0.04 event/min; *n* = 145 astrocytes; Fig. 3b). The proportion of hyperactive astrocytes was similar (*p* = 0.16) and the frequency distribution was not impacted by TTX application (Fig. 3d). In the same way, in individual patch-clamped astrocyte, the blockade of action potentials with TTX did not affect the Aβo effect on the proportion of active territories (*n* = 5 astrocytes from 4 animals; *p* = 1.0; Fig. 3f) and on the frequency of Ca^2+^ events (*n* = 870 ROIs; *p* = 0.9; Fig. 3g). The enlarged size of expanded events was not reduced with TTX (9.0 ± 0.5 μm^2^; *p* = 0.68; Additional file 4c). These data suggested that, within 5 min, Aβo acts directly on astrocyte signaling independently of any neuronal activity.

Activation of microglia can be a relatively early event in the Aβ mediated pathological process and can in turn activate astroglia cells [24, 25]. In order to assess the role of microglia in the Aβo-induced astrocytic Ca^2+^ hyperactivity, we pretreated slices with minocycline, an inhibitor of microglia activation [24–26]. Minocycline 50 nM pre-incubated during 15 min did not prevent the ability of Aβo to trigger astrocytic Ca^2+^ hyperactivity increasing to the same extent the proportion of active astrocytes (61.0 ± 3.3%; *n* = 6 slices from 4 animals; *p* = 0.0043) and the frequency of Ca^2+^ events (0.79 ± 0.08 event/min; *n* = 69 astrocytes; *p* = 0.02; Additional file 5a). Thus, within 5 min, Aβo acts directly on astrocyte signaling independently of any microglia activation.

To investigate the external and/or internal origin of Ca^2+^ implicated in the Aβo-induced Ca^2+^ hyperactivity, we applied 100 nM Aβo in Ca^2+^-free ACSF. Within the astrocytic population, we observed a 60% decrease of the proportion of active astrocytes (28.1 ± 2.7% active astrocytes in Ca^2+^-free medium versus 69.7 ± 2.1% in ACSF; *n* = 8 slices from 6 animals; *p* = 0.0002; Fig. 3a). The Aβo-induced frequency increase of Ca^2+^ events was also strongly reduced in Ca^2+^-free medium (0.30 ± 0.03 event/min; *n* = 73 astrocytes; *p* < 0.0001; Fig. 3b) and the proportion of hyperactive cells strongly declined (6% vs 41%; *p* < 0.0001; Fig. 3e). Within astrocytic processes, removing external Ca^2+^ avoided 1/3 of compartmentalized Aβo-induced Ca^2+^ hyperactivity (25.2 ± 5.2% of active territories; *n* = 5 astrocytes from 4 animals; *p* = 0.0016; Fig. 3f) and strongly reduced the frequency of the remaining signals (0.37 ± 0.01 event/min; *n* = 870 ROIs; *p* < 0.0001; Fig. 3g). The enlarged size of expanded events was however not significantly reduced in Ca^2+^-free medium (7.7 ± 0.7 μm^2^; *p* = 0.7; Additional file 4c). Overall, these data suggested that external Ca^2+^ entry is the main source of Aβo-induced Ca^2+^ hyperactivity in the astrocytic population and in the astrocytic processes, superimposing a new signal encoding.

### TRPA1 channels underlie Ca^2+^ hyperactivity induced by Aβo

**Fig. 4:**
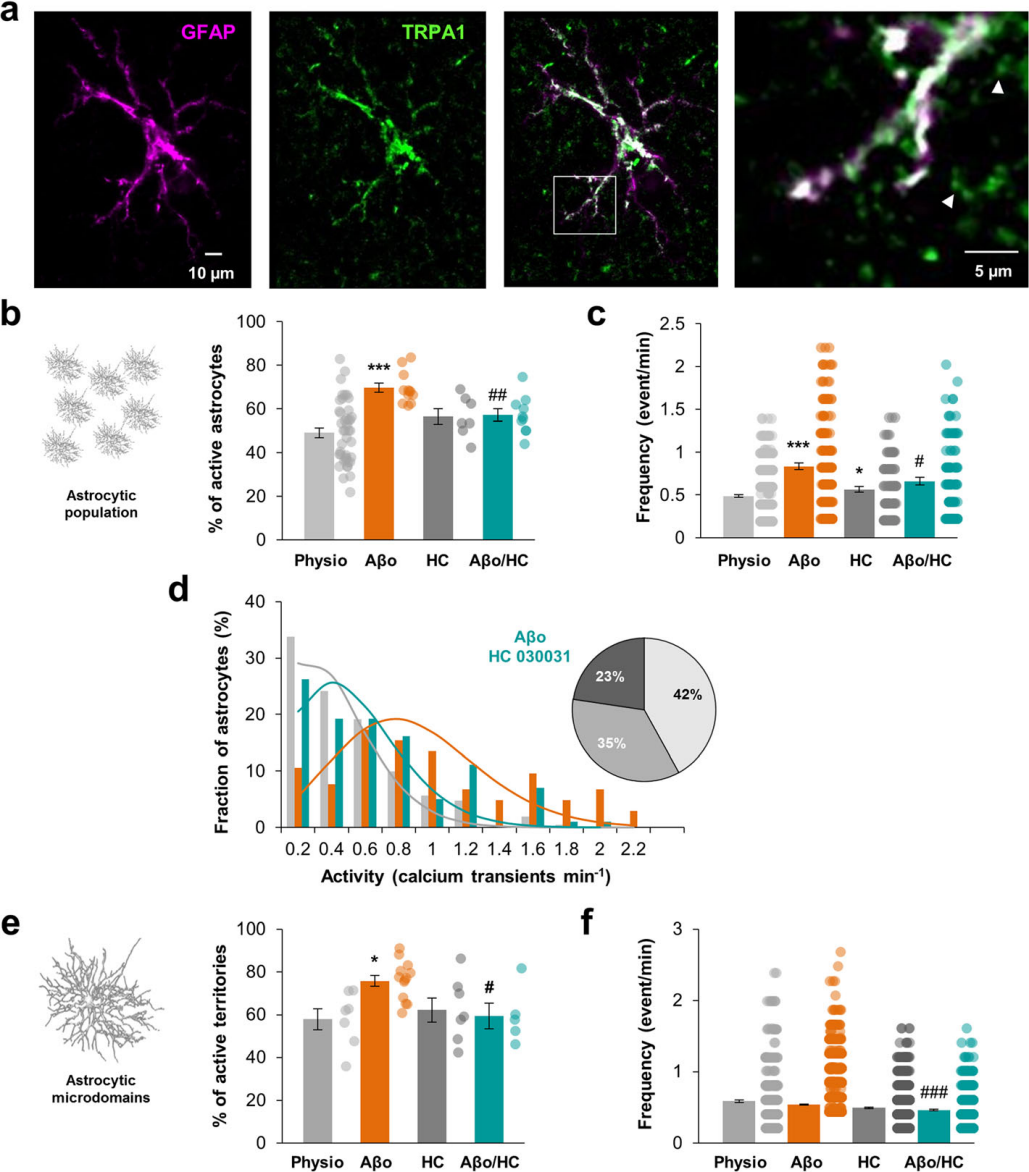
TRPA1 channels mediate astrocytic hyperactivity. **a** Immunohistochemistry of mouse *stratum radiatum* astrocytes showing that TRPA1 channels expression (*green*) was located in GFAP-positive processes (*magenta*) but also went over in distal processes excluding GFAP staining (e.g. *white arrowheads*). **b**, **c**. Within the astrocytic population, proportion of astrocytes displaying calcium activity and frequency of astrocyte calcium activity in physiological condition (*light grey*; *n* = 43), under 100 nM Aβo application (*orange*; *n* = 12), 40 μM HC 030031 application (*dark grey*; *n* = 7) and 100 nM Aβo + 40 μM HC 030031 co-application (*cyan*; *n* = 10). **d** Frequency histogram revealing that Aβo-induced hyperactivity is reduced when HC 030031 is co-applied (*cyan*). Related theoretical Poisson distributions were shown accordingly. **e**, **f**. Within single astrocyte arbor, proportion of subregions displaying calcium activity and frequency of astrocyte calcium activity in physiological condition (*light grey*; *n* = 7), under 100 nM Aβo application (*orange*; *n* = 13), 40 μM HC 030031 application (*dark grey*; *n* = 7) and 100 nM Aβo + 40 μM HC 030031 co-application (*cyan*; *n* = 5). Results are compared with the physiological condition with *, *p* < 0.05; **, *p* < 0.01 and ***, *p* < 0.001 or the Aβo condition with #, *p* < 0.05; ##, *p* < 0.01 and ###, *p* < 0.001


Within astrocyte, Ca^2+^ entry can occur through astrocytic ligand-gated Ca^2+^ channels (ionotropic receptors), transient receptor potential (TRP) receptors and reverse operation of Na^+^/Ca^2+^ exchanger (NCX). Astrocytic cationic ionotropic receptors (such as AMPA, NMDA or P2X receptors) have relatively low single channel conductance (~1 to 3 pS) and accounted for ~4% of fractional Ca^2+^ current [6]. Among TRP receptors, TRPA1 channels have been recently involved in regulating astrocyte basal Ca^2+^ levels in the hippocampus *stratum radiatum* [10, 11]. Interestingly, TRPA1 channels have a relatively high single channel conductance (~110 pS) accounting for ~17% of fractional Ca^2+^ current for the constitutively open channel [27] referring them as important actors involved in astrocytic Ca^2+^ entry. Within the CA1 region of mouse hippocampus, functional TRPA1 were only detected in astrocytes [10]. Based on immunohistochemistry of fixed brain sections, we found that TRPA1 channels staining appeared as discrete punctates predominantly co-localized with astrocytes (GFAP-positive cells; Additional file 6). Higher-resolution images showed that TRPA1 channels were expressed in both cell body and thick processes of *stratum radiatum* astrocytes objectivized by the GFAP co-staining. Some staining also appeared in adjacent territories (Fig. 4a) continuous with GFAP-positive stem processes. An additional 3D–reconstruct movie file shows this in more details (Additional file 7) and suggested that TRPA1 channels were expressed in astrocyte thin processes. These observations agreed with immunogold electron microscopy studies performed in rat trigeminal caudal nucleus showing that TRPA1 channels are localized in astrocyte peripheral processes [28].

Additional file 7: TRPA1 channels expression is located in thick GFAP-positive processes and in adjacent thin processes lacking GFAP staining. 3D–recontsruct of astrocytic processes objectivized by GFAP staining (magenta) showing that TRPA1 channels (green) expression went over in contiguous processes excluding GFAP staining. Scale bar: 50 nm. (MP4 25.2 mb)


In physiological condition, incubation of slices with the selective TRPA1 blocker, HC 030031 (40 μM) [29], during 5 min had no effect on the proportion of spontaneous active astrocytes within the global astrocytic population (56.5 ± 3.5% active astrocytes; *n* = 7 slices from 3 animals; *p* = 0.17; Fig. 4b) but increased the frequency of Ca^2+^ events (0.56 ± 0.03 event/min; *n* = 167 astrocytes; *p* = 0.043; Fig. 4c). In patch-clamped cells, incubating 5 min slices with 40 μM HC 030031 had no impact on the proportion of active microdomains (62.8 ± 5.7%; *n* = 7 astrocytes from 4 animals; *p* = 0.71; Fig. 4e) or on Ca^2+^ signal frequency (0.50 ± 0.01 event/min; *n* = 919 ROIs; *p* = 0.19; Fig. 4f). Together, these data confirmed that TRPA1 does not massively contribute to spontaneous Ca^2+^ signaling in the astrocytic arbor territory of *stratum radiatum* astrocytes [11, 30].

Interestingly, when Aβo was applied in presence of HC 030031 (40 μM), the astrocytic Ca^2+^ hyperactivity was totally abolished and restored to basal levels either in the global astrocytic population or in the processes territory. In bulk loading condition, the proportion of active astrocytes decreased (57.1 ± 2.8% in Aβo + HC 030031 vs 69.7 ± 2.1% in Aβo condition; *n* = 10 slices from 3 animals; *p* = 0.0021; Fig. 4b) and was reversed to physiological condition level (49.1 ± 2.2%; *p* = 0.08). Frequency also decreased when HC 030031 was co-applied with Aβo (0.67 ± 0.04 event/min vs 0.83 ± 0.04 event/min in Aβo condition; *n* = 181 astrocytes; *p* = 0.013; Fig. 4c) but remained higher than in physiological condition (0.49 ± 0.01 event/min; *p* = 0.0002). Frequency distribution tended toward physiological distribution and the proportion of hyperactive astrocytes was reduced (23% in Aβo + HC 030031 vs 41% in Aβo condition; *p* = 0.023; Fig. 4d). Within individual cells, the proportion of active microdomains declined (59.4 ± 6.0% of active microdomains in Aβo + HC 030031 vs 75.8 ± 2.5% in Aβo condition; *n* = 5 astrocytes from 4 animals; *p* = 0.03) close to physiological value (57.9 ± 4.8%; *p* = 0.87; Fig. 4e) together with the events frequency (0.47 ± 0.09 event/min in Aβo + HC 030031 vs 0.54 ± 0.07 event/min in Aβo condition; *n* = 760 ROIs; *p* < 0.0001; Fig. 4f). The ratio of expanded/focal event decreased (30% of expanded event in Aβo + HC 030031 vs 41% in Aβo condition; *p* = 0.039; Additional file 4a) reaching physiological value (33%; *p* = 0.45). Expanded surface extension also shrank (7.2 ± 0.5 μm^2^ vs 9.2 ± 0.5 μm^2^; *p* = 0.017; Additional file 4c) close to physiological size (7.1 ± 0.4 μm^2^).

Thus, while having a negligible impact on physiological spontaneous Ca^2+^ activity, blocking TRPA1 channels cancelled astrocyte Ca^2+^ hyperactivity induced by Aβo leading most of the spatiotemporal Ca^2+^ signal properties back to physiological state in either the astrocytic arbor microdomains or the global astrocytic population.

### APP/PS1–21 mice display early astrocytic hyperactivity that involves TRPA1 channels

**Fig. 5:**
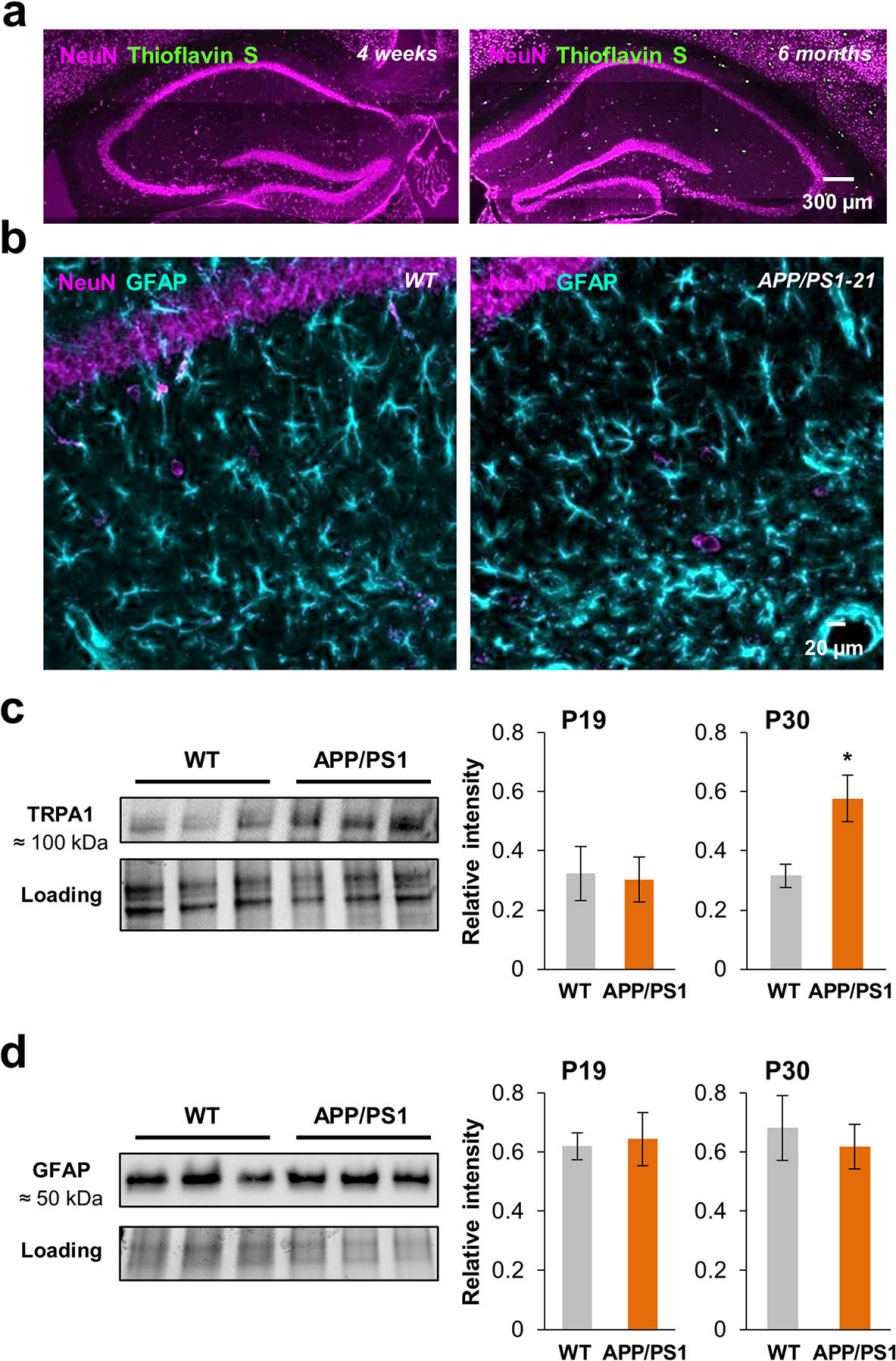
Young APP/PS1–21 mice are devoid of amyloid deposit and reactive astrocyte whereas gradually overexpress TRPA1. **a** Thioflavin S staining for β-amyloid deposits (*green*) and NeuN immunostaining (*magenta*) in the hippocampus of 1-month-old (*left*) and 6-month-old (*right*) APP/PS1–21 mice showing the progression of the number of amyloid deposits. **b** GFAP (*cyan*) and NeuN (*magenta*) immunostainings in the *stratum radiatum* of a P30 WT (*left*) and APP/PS1–21 (*right*) mice. **c** Western-blot analysis of protein levels of TRPA1 channels in hippocampus extracts from P19 and P30 WT and APP/PS1–21 mice (3 different extracts of P30 WT and APP/PS1–21 mice are shown). Histogram showing quantification of TRPA1 channels expression normalized to protein loading levels (*n* = 6 hippocampus in each group at P19 and 8 hippocampus in each group at P30). **d** Western-blot analysis of protein levels of GFAP in hippocampus extracts from P19 and P30 WT and APP/PS1–21 mice (extracts of same P30 lysates depicted in c are shown). Histogram showing quantification of GFAP expression normalized to protein loading levels (*n* = 7 hippocampus in each group at P19 and P30). Results are compared with the WT mice with *, *p* < 0.05; **, *p* < 0.01 and ***, *p* < 0.001

APP/PS1–21 mice co-express the human KM/67/671NL mutation in the amyloid precursor protein (APPswe) and the human L166P-mutated presenilin 1 (PS1) under the control of Thy1 promotor. These transgenic mice display high transgene expression with Aβ_1–42_ levels being significant at 1 month and plaques appearing at 6 weeks of age in the neocortex and at 4–5 months in the CA1 hippocampus [15]. As external application of Aβo induced an immediate astrocytic hyperactivity in hippocampal healthy brain slices, we wonder about the effect of Aβ in APP/PS1–21 mice at the beginning of its secretion within the brain. Thy1 is known to be expressed from ~P14 in mouse CA1 hippocampus [31] thus Aβ_1–42_ might be produced from this stage. However, while being present at this early stage [15], Aβ were not yet aggregated in plaques in APP/PS1–21 mice at 1-month-old (Fig. 5a) while Aβ plaques were present in the hippocampus at 6-month-old as testified with Thioflavin S labeling [21]. Likewise, astroglial marker such as GFAP showed a similar profile in WT versus APP/PS1–21 mice at 1-month-old confirming the lack of reactive astrogliosis in these early stages (Fig. 5b, d). Thus, studying astrocyte activity at these early stages allowed us to measure the impact of not aggregated Aβ on healthy astrocytes in an AD transgenic mice model.

We first investigated the expression of TRPA1 channels in the hippocampus of young APP/PS1–21 mice and their littermates (WT). We found that the protein level of TRPA1 channels was similar in P19 mice (*n* = 6 hippocampus from 3 animals for each; *p* = 0.9; Fig. 5c) and twice higher in P30 APP/PS1–21 than WT mice (*n* = 8 hippocampus from 4 animals for each; *p* = 0.02; Fig. 5c) suggesting an early upregulation of this channel from the onset of Aβ secretion. At the same time, GFAP protein level was stable (Fig. 5d) testifying the lack of astrogliosis in these early AD stages.

**Fig. 6:**
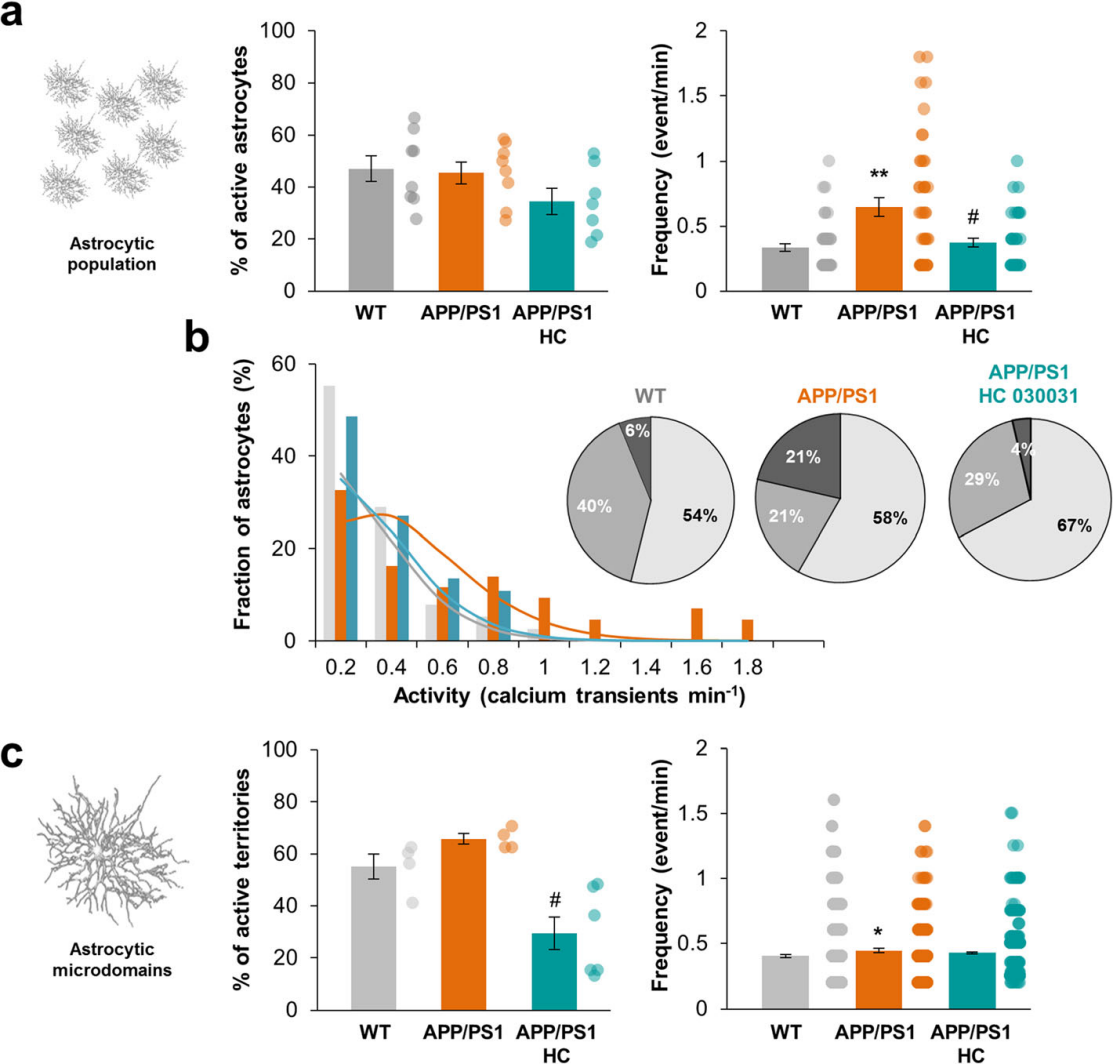
Young APP/PS1–21 mice display an early global and compartmentalized astrocytic hyperactivity. **a** Within the global astrocytic population, proportion of astrocytes displaying calcium activity and frequency of calcium activity in APP/PS1–21 mice (*orange*; *n* = 8) and their littermates (WT, *grey*; *n* = 8) in physiological condition or with 40 μM HC 030031 (*cyan*, *n* = 7). **b** Frequency histogram showing the occurrence of Ca^2+^ events in the astrocyte population of APP/PS1–21 (*orange*), WT (*grey*) and APP/PS1–21 mice treated with 40 μM HC 030031 (*cyan*). Related theoretical Poisson distributions were shown accordingly. Pie chart representing inactive, active and hyperactive astrocytes in WT and APP/PS1–21 mice treated or not with HC 030031. **c** Within single astrocyte arbor, proportion of subregions displaying calcium activity and frequency of calcium activity in APP/PS1–21 mice (*orange*; *n* = 4) and their littermates (WT, *grey*; *n* = 4) in physiological condition or with 40 μM HC 030031 (*cyan*, *n* = 7). Results are compared with the WT mice with *, *p* < 0.05; **, *p* < 0.01 and ***, *p* < 0.001 or the APP/PS1–21 mice with #, *p* < 0.05; ##, *p* < 0.01 and ###, *p* < 0.001

We then recorded astrocytic Ca^2+^ signals in young APP/PS1–21 mice (from P19 to P28) and compared to their littermates (WT). In bulk loaded slices, the proportion of active astrocytes within *stratum radiatum* was similar in both condition (45.4 ± 4.1% in APP/PS1–21 vs 47.1 ± 4.9% in WT; *n* = 8 slices from 5 animals in each condition; *p* = 0.8; Fig. 6a). However, the frequency of Ca^2+^ events was significantly higher in APP/PS1–21 cells (0.65 ± 0.07 event/min in APP/PS1–21 vs 0.33 ± 0.03 event/min in WT; *n* = 103 and 105 astrocytes respectively; *p* = 0.0013; Fig. 6a) and the frequency distribution was switched to higher frequency in APP/PS1–21 compared to WT mice (λ = 2.1 vs 1.21 in WT; Fig. 6b) leading to an increase of the fraction of hyperactive cells (21% vs 6% in WT; *p* = 0.0009; Fig. 6b).

In this AD mouse model, no signs of neuroinflammation were apparent in 1-month-old mice and microgliosis has been described to be concomitant with plaques appearance [15]. Indeed, at these early stages, microglia phenotype was not different in APP/PS1–21 mice from WT littermate (Iba-1 immunostaining; Additional file 5b). However, to confirm that microglia was not involved in these early astrocytic events, we pretreated slices with minocycline in order to inhibit potential microglia activation [24–26]. Minocycline 50 nM, pre-incubated during 15 min, had no effect on either the proportion of active cells or the frequency of astrocytic Ca^2+^ events within APP/PS1–21 mice (*n* = 6 slices from 4 animals; *p* = 0.88 and 0.82 respectively; Additional file 5c) testifying that microglia activation was not involved in the astrocytic hyperactivity setting up at the beginning of Aβ secretion in this AD mouse model. In patch-clamp loaded astrocyte, the proportion of active microdomains increased in APP/PS1–21 mice (65.7 ± 2.0% vs 55.0 ± 4.8% in WT; *n* = 4 astrocytes from 4 animals for each; *p* = 0.052; Fig. 6c) together with the frequency of events within these microdomains (0.45 ± 0.02 event/min vs 0.40 ± 0.01 event/min in WT; *n* = 255 and 426 ROIs respectively; *p* = 0.04; Fig. 6c). The proportion of expanded/focal events was slightly affected in APP/PS1–21 mice in favor of expanded event and the size of expanded events was similar (6.8 ± 0.3 μm^2^ in WT vs 6.7 ± 0.3 μm^2^ in APP/PS1–21; *p* = 0.42; Additional file 4b, d). Thus, in young APP/PS1–21 mice, Ca^2+^ activity in the global astrocytic population and the compartmentalized activity within the astrocytic arbor started to be affected from the beginning of Aβ overproduction.

In these conditions, blocking TRPA1 channels with HC 030031 (40 μM) in APP/PS1–21 mice strongly decreased the frequency of Ca^2+^ events (0.37 ± 0.03 event/min with HC 030031 vs 0.64 ± 0.07; *n* = 113 astrocytes from 7 slices; *p* = 0.013; Fig. 6a) resulting in a clear redistribution of Ca^2+^ events frequency (λ = 1.35) and a decrease of the proportion of hyperactive cells that look alike the WT situation (4% in APP/PS1–21 + HC 030031 vs 21% in APP/PS1–21; *p* = 0.0025; Fig. 6b). In patch-clamp loaded astrocyte, HC 030031 induced a significant decrease of the proportion of active microdomains in APP/PS1–21 mice (29.3 ± 6.2%; *n* = 7 from 4 animals; *p* = 0.01; Fig. 6c) together with a restrained effect on the frequency (0.43 ± 0.06 event/min; *n* = 1213 and 1577 ROIs respectively; *p* = 0.058). The proportion of expanded/focal events was also reduced with HC 030031 treatment (Additional file 4b). Overall, these observations in young APP/PS1–21 mice matched the above related effect of Aβo application in healthy slices consistently with a TRPA1 channel involvement. Merely, they were more subtle and initially restrained to the astrocytic arbor.

### Aβo-induced CA1 neurons hyperactivity is related to TRPA1 channels activation

**Fig. 7:**
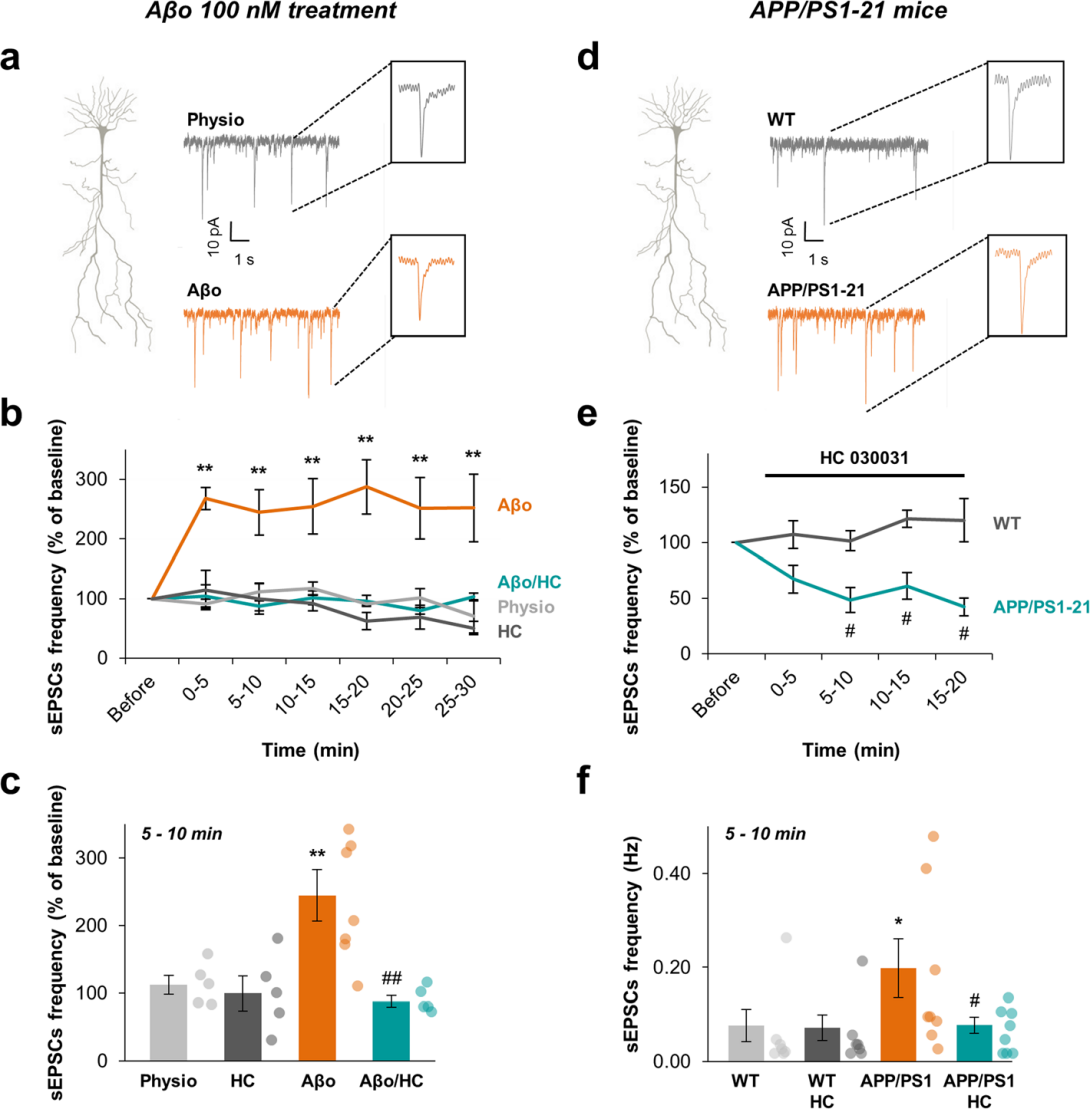
Aβo increases the frequency of spontaneous excitatory post-synaptic currents (sEPSCs) from CA1 pyramidal neurons in a TRPA1 channels dependent manner. **a** Representative traces of voltage-clamp recordings from CA1 pyramidal cells held at −65 mV in physiological condition (*grey*) and under Aβo 100 nM application (*orange*). Examples of single EPSC with higher time resolution are shown in the corresponding insets. **b** Time course of the frequency of sEPSCs in physiological condition (*light grey*; *n* = 5), under application of 100 nM Aβo (*orange*; *n* = 7), 40 μM HC 030031 (*dark grey*; *n* = 5) or co-application of 100 nM Aβo + 40 μM HC 030031 (*cyan*; *n* = 4). **c** Histogram showing the sEPSCs frequency at a time matching the astrocyte calcium activity measurements (i.e. 5 to 10 min after drugs application). Results are compared with the physiological condition with *, *p* < 0.05; **, *p* < 0.01 and ***, *p* < 0.001 or the Aβo condition with #, *p* < 0.05; ##, *p* < 0.01 and ###, *p* < 0.001. **d** Representative traces of voltage-clamp recordings from CA1 pyramidal cells held at −65 mV in APP/PS1–21 mice (*orange*) and their WT littermates (*grey*). Examples of single EPSC with higher time resolution are shown in the corresponding insets. **e** Time course of the normalized frequency of sEPSCs in WT (*dark grey*; *n* = 7) and APP/PS1–21 mice (*cyan*; *n* = 8) under application of 40 μM HC 030031. **f** Histogram showing the sEPSCs frequency in basal condition in WT (*light grey*; *n* = 7), APP/PS1–21 mice (*orange*; *n* = 8) and 5 to 10 min after HC 030031 application (*dark grey* and *cyan* respectively). Results are compared to the WT basal activity with *, *p* < 0.05; **, *p* < 0.01 and ***, *p* < 0.001 or the APP/PS1–21 basal activity with #, *p* < 0.05; ##, *p* < 0.01 and ###, *p* < 0.001

Astrocytes are known to actively regulate synaptic activity and plasticity [4]. Given the time range of the Aβ-mediated impact on astrocyte excitability, we wonder about its consequences on basal synaptic activity. Thus, to explore the outcome of Aβo induced TRPA1-dependent astrocyte hyperexcitability on neighboring synapses, we recorded CA1 pyramidal neurons spontaneous excitatory post-synaptic currents (sEPSCs) performing whole-cell patch-clamp recordings on CA1 neurons cell bodies (Fig. 7a). The initial mean frequency of sEPSCs recorded from CA1 pyramidal neurons was 0.13 ± 0.03 Hz (*n* = 22 neurons from 17 animals) and was stable during a 30-min recording (Fig. 7b). The mean peak amplitude of sEPSCs recorded from CA1 pyramidal neurons was 31.1 ± 2.0 pA. Interestingly, application of 40 μM HC 030031 alone had no effect on sEPSCs frequency (74 ± 22% from 5 to 10 min; *n* = 5 cells from 3 animals; *p* = 0.6; Fig. 7b, c) suggesting that TRPA1 channels were not involved in physiological sEPSCs modulation. Application of 100 nM Aβo in the extracellular medium induced a rapid, strong and persistent increase of sEPSCs frequency (Fig. 7b; *n* = 7 cells from 6 animals) with no effect on the sEPSCs amplitude (32.4 ± 2.5 pA; *p* = 0.3). We confirmed that application of monomeric forms of Aβ_1–42_ (Aβm) had no effect on sEPSCs frequency during the 30-min recording (*n* = 5; *p* = 0.78; Additional file 1d) settling that only oligomeric forms of Aβ_1–42_ were active. Interestingly, the strong and persistent increase of sEPSCs frequency induced by Aβo was time-concomitant with the setting up of astrocyte hyperexcitability (i.e. 5 to 10 min, 245 ± 38%; *p* = 0.01; Fig. 7c) and was fully abolished by a co-application of HC 030031 (40 μM) with Aβo (88 ± 8% from 5 to 10 min; *n* = 5 cells from 5 animals; *p* = 0.005; Fig. 7b, c). This suggests that TRPA1 channels-dependent astrocyte hyperactivity induced by Aβo directly influenced CA1 neurons activity and its blockade was enough to counteract the neuronal hyperactivity, at least in the short and medium term.

We then recorded spontaneous EPSCs in APP/PS1–21 mice at the onset of Aβ overproduction (P20-P28) and compared to their littermates (WT). We observed an increase of CA1 neurons sEPSCs frequency setting up at P20 in APP/PS1–21 mice (0.20 ± 0.06 Hz in APP/PS1–21 vs 0.08 ± 0.03 Hz in WT; *n* = 8 neurons from 6 animals in APP/PS1–21 and 7 neurons from 5 animals in WT; *p* = 0.0271; Fig. 7d, f) with no difference in their amplitude (33.7 ± 1.9 pA in APP/PS1–21 vs 33.0 ± 2.3 pA in WT; *p* = 0.955). These data confirmed that neuronal hyperexcitability is one of the earliest dysfunction in the pathophysiological cascade initiated by abnormal Aβ accumulation [32]. Application of HC 030031 (40 μM) strongly reduced sEPSCs frequency in APP/PS1–21 mice as soon as 5 min after its bath perfusion (0.08 ± 0.02 Hz; *n* = 8 neurons from 6 animals; *p* = 0.0234) while it had no effect on WT mice (0.07 ± 0.03 Hz; *n* = 7 neurons form 5 animals; *p* = 0.854; Fig. 7e, f). These data corroborate the highlighted precocious involvement of TRPA1 channel in AD pathogenesis.

## Discussion

In this study, we investigated the contribution of astrocytes in early Aβo toxicity by studying calcium signaling in different parts of the whole astrocyte territory. We found that astroglia is a frontline target of Aβo exhibiting a global and local Ca^2+^ hyperactivity that involves TRPA1 channels. This TRPA1 channel-dependent astrocytic Ca^2+^ hyperactivity exerts regulatory influences on synaptic function and is linked to the glutamatergic synapse hyperactivity recorded in CA1 neurons. Concurrently to these acute Aβo-induced effects, astrocytes in young APP/PS1–21 mice hippocampus elicit a similar pattern of calcium hyperactivity in close relationship with the setting up of a precocious neuronal hyperactivity that are both reversed when TRPA1 channel is blocked. Moreover, the TRPA1 channel is gradually overexpressed at the onset of Aβ production in this AD mouse model.

Intracellular Ca^2+^ transients are considered as the primary signal by which astrocytes interact with each other and with neighboring neurons. Ca^2+^ has been extensively studied within the astrocytic cell body and thick branches. More recently, local Ca^2+^ dynamics in distal fine processes has been investigated emphasizing a highly compartmentalized signaling, interconnected with physiological transmission at neighboring synapses [8, 9]. Compartmentalization of astrocytic Ca^2+^ dynamics needs to be attentively considered in order to understand how astrocytes may contribute to brain information processing [8]. We thus chose to study both levels of information (i.e. global population signaling and local microdomain signaling) combining bulk loading and single cell astrocyte loading. Genetically encoded Ca^2+^ indicators (GECIs) have been recently used to study Ca^2+^ signals in distal thin processes [9]. Alternatively, patch pipette loading give access to the whole territory of a single astrocyte and, currently, Fluo-4 is far more sensitive than GECIs therefore enabling to track smaller signals [8]. Accordingly, we observed a similar and even better diffusion of Fluo-4 in a single astrocyte compared to SR101. We first characterized the physiological calcium activity of mouse CA1 *stratum radiatum* astrocytes and showed that this activity is fully autonomous, i.e. independent of neuronal activity, both at the astrocytic population level and at the microdomain level. This is in agreement with data obtained in mouse CA1 hippocampus [30, 33] but not with astrocyte behavior in the dentate gyrus where expanded Ca^2+^ events were partly dependent on neuronal activity [8]. Interestingly, external Ca^2+^ entry is the main source of Ca^2+^ within thin processes whereas it only partly contributes to somatic signaling. This discrepancy between Ca^2+^ sources in astrocyte soma and distal processes has already been described in brain slices [34]. This might be supported by the subcellular location of calcium stores that are concentrated in the cell body and thick processes but are almost absent from thin processes [23].

A central element of the pathogenesis of AD is the progressive accumulation of Aβo species, ultimately resulting in the formation of plaques. Yet, small soluble Aβ oligomers are sufficient to induce several features of the AD phenotype [1]. The paths by which Aβo leads to neurodegeneration are probably multifactorial but all converge all towards synaptic dysfunction. The major challenge of AD research is to understand the complex cellular reaction underlying the long prodromal phase of AD [14]. Astrocytes are an integral part of synaptic transmission and are therefore critical for the establishment and maintenance of neuronal health [5]. They contribute to neuronal dysfunction by being proinflammatory [35] but also play a protective role, e.g. through the release of gliotransmitters [36] and Aβ clearance [37, 38]. It is therefore of major importance to distinguish the beneficial from the deleterious impact of Aβo on astrocyte function. Aβo has already been involved as a direct effector on astrocytes in primary cultures [39], in hippocampal slices [40] and in vivo [12] but here we showed a peculiar rapid action on compartmentalized calcium activity, activating a membrane Ca^2+^ permeable channel. Indeed, Aβo application triggers global Ca^2+^ hyperactivity in CA1 hippocampus astroglial population together with an intensification of the compartmentalized Ca^2+^ activity in the astrocytic processes and a spatial extension of the size of the expanded Ca^2+^ events within microdomains. These effects are specific to oligomeric forms of Aβ since application of the monomeric form in the same conditions had no impact on astrocytic calcium activity. We reported that the effect of Aβo on astrocyte excitability is fully independent of neuronal activity since TTX application does not prevent the Aβo effect neither on the global hyperactivity nor on the compartmentalized hyperactivity in processes. Concurrently, microglia activation does not participate in this astrocytic hyperactivity, at least in the time scale studied, whereas longer applications of Aβ activate microglia together with astroglia [24]. Thus, astrocytes seem to express a distinctive precocious detector involved at the onset of Aβo appearance. Removal of external Ca^2+^ largely inhibits Aβo-induced astrocyte hyperactivity at the population level while it has no effect in physiological conditions. Removal of external Ca^2+^ also inhibits the majority of the compartmentalized Aβo-induced hyperactivity. Thus, transmembrane Ca^2+^ entry carries most of the Aβo-induced hyperactivity. Remarkably, we showed that both global and compartmentalized hyperactivities are driven by TRPA1-dependent Ca^2+^ entry since HC 030031, a specific TRPA1 channel inhibitor [29], strongly abolishes Aβo-induced astrocyte hyperexcitability and totally restores the spatiotemporal properties of Ca^2+^ events back to a physiological level.

The TRPA1 channel is a Ca^2+^ permeable non-selective cation channel initially known to be expressed in primary afferent nociceptive neurons [29]. In mouse CA1 hippocampus, TRPA1 channels are found to be preferentially expressed in astrocytes [10]. However, their involvement in physiological astrocytic Ca^2+^ signaling is highly debated. It has been shown that TRPA1 channels contribute to maintain basal Ca^2+^ levels and regulate ~20% of spontaneous Ca^2+^ signals within astrocyte branches [11] but in the end, poorly take part in basal astrocytic Ca^2+^ signaling [30, 33]. Our data assume that TRPA1 channels are only slightly involved in the astrocytic Ca^2+^ signaling in physiological conditions. However, we highlighted they are quickly and largely involved in case of Aβo presence. The absence of an obvious involvement of these channels in astrocyte physiological Ca^2+^ signaling is startling since we evidenced a TRPA1 channel expression in thick and in adjacent thin astrocytic processes. Thus, TRPA1 channels might only behave as an “aggression sensor”. Indeed, TRPA1channel gating is particularly regulated by numerous electrophilic activators - such as reactive oxygen species, reactive nitrogen species or oxidized lipids - and also functions as a mechanosensor [27]. Hence, TRPA1 channels might be directly targeted by Aβo or might be secondarily activated through an Aβo-induced oxidative stress and/or through its mechanosensor properties if Aβo binds to the astrocytic cholesterol-rich plasma membrane [14].

Strikingly, in young APP/PS1–21 mice (~3–4 weeks), we observed a similar pattern of astrocytic hyperactivity starting at the beginning of Aβ overproduction in the hippocampus, long before its aggregation into plaques. These early repercussions in young APP/PS1–21 mice were restricted to the frequency of astrocytic Ca^2+^ events in either the astrocytic population and microdomains of astrocytic processes with fewer impacts on the proportion of active cells or microdomains. This suggests a gradual impact of surrounding Aβ on astrocyte signaling, increasing the frequency of compartmentalized Ca^2+^ events and, to a lesser extent, the proportion of active territories within the astrocytic processes. These impacts on astrocytic processes go along with a noteworthy redistribution of the frequency of Ca^2+^ events within the astrocytic population. Interestingly, blockade of TRPA1 channels with HC 030031 abolished the astrocyte Ca^2+^ hyperactivity. Overall, TRPA1 channel signaling seems to be at the frontline in mediating these Aβo progressive effects in early stages of AD. Data obtained in an advanced AD transgenic model showed an astrocyte network hyperactivity in cortical areas close to Aβ plaques and an involvement of metabotropic purinergic signaling in this astrocyte hyperactivity [13]. This suggests a differential evolution of astrocyte engagement in AD pathogenesis depending on the stage, the structure and the physiopathological state of the astrocyte. Likewise, it has been reported that the TRPA1 channels’ protein level was increased in hippocampal astrocytes of 8 month-old APP/PS1 mice at where it mediated inflammation through astrocyte activation [41]. Here, we showed that TRPA1 channel expression in hippocampus is increased much earlier, as soon as 1 month of age, in a more aggressive AD mouse model (APP/PS1–21). These data point towards a TRPA1 channel contribution in early stages of pathophysiology, that is as soon as the Aβo level increases and long before the setting up of astrogliosis or inflammatory mechanisms.

Numerous laboratory studies in the past decade have shown that Aβo impairs synaptic function and synaptic structure [42]. However, how soluble Aβo initiates these effects remains to be determined. Each astrocyte deploys many fine processes to contact up to 140,000 synapses in the CA1 region [43]. As we highlighted an intense and early effect of Aβo on astrocyte Ca^2+^ activity within processes, we assessed the link with spontaneous neuronal activity. Indeed Aβo is also known to enhance spontaneous neuronal excitability in CA1 [32, 44, 45]. Consistently, we showed here that Aβo induces a rapid and strong increase of spontaneous EPSCs frequency in CA1 neurons. Strikingly, blocking TRPA1 channels totally prevents this Aβo neuronal impact. Yet, when we blocked neuronal activity with TTX, we did not affect the Aβo-induced astrocyte hyperactivity which would be partly the case if a neuronal TRPA1 was involved. This precocious Aβo impact thus seems to trigger a one-way communication from astrocyte to neuron related to TRPA1 activation. This TRPA1-dependent neuronal hyperactivity was similarly observed in APP/PS1–21 mice at the onset of Aβ overproduction testifying its physiopathological relevance in the AD initiation process. It has been shown that Aβ can increase astrocytic release of glutamate to the extrasynaptic space resulting in the activation of extrasynaptic NMDARs and the disruption of neuronal signaling [40, 46, 47]. Besides, astrocytes can regulate synaptic and extrasynaptic neurotransmitter concentrations, such as glutamate, in a Ca^2+^-dependent manner e.g. via vesicular release, bidirectional transport or hemichannel opening [48]. We will further decipher pathways implemented by the Aβ-induced TRPA1-mediated Ca^2+^ entry that consequently affect neuronal transmission.

It has been demonstrated that soluble Aβo can affect astrocyte signaling properties in various ways in mouse hippocampal CA1 astrocytes [40, 49, 50]. To some extent, the involvement of TRPA1 channels superimposed to these effects, directly affecting local synaptic function in a distinctive precocious manner. This actor might thus contribute to the complex cellular phase of AD, upstream of symptomatic neurodegeneration [14].

## Conclusions

In this work, we have shown that intricate global and compartmentalized astroglial Ca^2+^ signaling disturbances induced by Aβo are mediated by a TRPA1 channel-dependent Ca^2+^ signaling. This new highlighted mechanism is at work with both external supply of Aβo and at the onset of Aβ production in an AD transgenic mouse model. This astrocytic pathway is promptly implemented and involved in the well-known and characteristic Aβo-induced synaptic dysfunction. Blockade of TRPA1 channel, that appears to be preferentially expressed in astrocytes within the hippocampus [11], is sufficient to counteract the impact of Aβo on spontaneous neuronal activity. This suggests that astrocytes can be considered as a particularly precocious target in Aβo toxicity consequently affecting nearby synapses. Up until now, altered astrocyte activation was usually associated with late AD, i.e. when amyloid plaques are already developed, and the role of astrocytes in the initial toxic effect of Aβo was not yet evidenced. One of the major findings of our study is to suggest that astrocytes are implicated far before astrogliosis and inflammatory processes. Thus, focusing on this astrocyte involvement, for instance by modulating TRPA1 channel activity, may represent a novel target to hamper early dysfunction in AD.

## Additional files

Additional file 1: Characterization and effects of Aβ oligomers and purified monomers. (**a**) SDS-PAGE analysis of Aβ oligomers examined by western-blot with 4G8 antibody. Aβo showed faintly monomers and abundant dimers and trimers. (**b**) SDS-PAGE analysis of Aβ monomers after purification on C18 column. All fractions were electrophoresed on 15% tris-glycine gel. Aβ monomer is mainly eluted at 30% acetonitrile. S, sample loaded; FT, flow through; peptides eluted at 30, 40, 50 and 60% acetonitrile. **(c)** Within the astrocytic population, proportion of astrocytes displaying calcium activity and frequency of astrocyte calcium activity in physiological condition (grey; *n* = 43), under 100 nM Aβo application (orange; *n* = 12) and under 100 nM Aβm application (light orange; *n* = 7). (**d**) Time course and histogram at 5–10 min of the frequency of sEPSCs in physiological condition (grey; *n* = 5), under application of 100 nM Aβo (orange; *n* = 7) or 100 nM Aβm (light orange; *n* = 5). In any case, Aβm was obtained from the 30% acetonitrile fraction purified in b. (TIFF 291 kb)

Additional file 2: Characterization of Fluo-4-loaded cells in the *stratum radiatum* of mouse coronal slice. (**a**) Confocal image of Fluo-4-loaded (cyan) and SR101-labeled (magenta) cells in the CA1 *stratum radiatum*. Merged image showing the proportion of loaded astrocytes (white), confirming that most of the loaded cells are astrocytes. One hour before slicing, animals were iv injected with SR101 as described previously [51]. Vessels are only labeled with SR101 (white arrow). (**b**) Z-stack projections of confocal images of a patched astrocyte loaded with Fluo-4 (cyan) and SR101 (magenta). Merged image showing the Fluo-4 diffusion in the whole astrocytic territory. (**c**) Example of a passive whole-cell current recorded in a *stratum radiatum* astrocyte. Cell was held at −70 mV and 10 mV hyper- and depolarizing voltage steps of 80 ms duration were applied (−110 to +80 mV). (TIFF 1907 kb)

Additional file 4: Spatial properties of compartmentalized calcium events in the astrocytic processes. (**a**) Proportion of expanded/focal events in physiological condition, under 100 nM Aβo application, 100 nM Aβo + 500 nM TTX co-application and 100 nM Aβo + 40 μM HC 030031 co-application. (**b**) Proportion of expanded/focal events in APP/PS1–21 mice and their littermates (WT) in physiological condition or under 40 μM HC 030031 treatment. (**c**) Mean size of expanded Ca^2+^ events in physiological condition (grey), under 100 nM Aβo application (orange), 100 nM Aβo + 500 nM TTX co-application (dark blue), 40 μM HC 030031 (dark grey), 100 nM Aβo in Ca^2+^-free medium application (0 Ca; light blue) and 100 nM Aβo + 40 μM HC 030031 co-application (cyan). (**d**) Mean size of expanded Ca^2+^ events in APP/PS1–21 mice (orange) and their littermates (WT, light grey) in physiological condition or with 40 μM HC 030031 treatment (WT, dark grey and APP/PS1–21, cyan). Results are compared with the physiological condition with *, *p* < 0.05; **, *p* < 0.01 and ***, *p* < 0.001 or the Aβo condition with #, *p* < 0.05; ##, *p* < 0.01 and ###, *p* < 0.001. (TIFF 752 kb)

Additional file 5: Blockade of microglia activation with minocycline does not prevent the effect of Aβo on astrocyte Ca^2+^ activity. (**a**) Within the astrocytic population, proportion of astrocytes displaying calcium activity and frequency of astrocyte calcium activity in physiological condition (grey; *n* = 43), under 50 nM minocycline (dark grey; *n* = 5); 100 nM Aβo application (orange; *n* = 12) and under 50 nM minocycline +100 nM Aβo application (dark orange; *n* = 6). (**b**) Immunohistochemistry of mouse *stratum radiatum* microglia showing that Iba1-positive cells were not hypertrophic in one-month-old APP/PS1–21 mice when compared to WT littermates. Higher magnification of representative microglia is shown in the lower panels. (**c**) Proportion of astrocytes displaying calcium activity and frequency of astrocyte calcium activity in APP/PS1–21 mice (orange; *n* = 8), under 50 nM minocycline application (dark orange; *n* = 6) or in WT littermates (grey; *n* = 8). Minocycline is pre-incubated 15 min before recording. Results are compared with the physiological condition, with or without minocycline, or with the WT littermates with *, *p* < 0.05; **, *p* < 0.01 and ***, *p* < 0.001. (TIFF 1680 kb)

Additional file 6: TRPA1 is expressed in *stratum radiatum* astrocyte cell body and processes. (**a**) Immunohistochemistry of mouse *stratum radiatum* astrocytes showing that TRPA1 channels (green) are expressed within astrocytic domains certified by GFAP staining (magenta). (**b**) Merge image showing that most TRPA1 staining co-localized and surrounded GFAP-positive processes. (TIFF 2786 kb)

## Abbreviations

AD: Alzheimer’s disease
APP: Amyloid Precursor Protein
Aβ: Amyloid-β peptide
Aβm: Amyloid-β monomers
Aβo: Amyloid-β oligomers
Ca^2+^: calcium
CA1: Cornu Ammonis 1
GFAP: Glial Fibrillary Acidic Protein
PS1: Presenilin 1
ROI: Regions Of Interest
sEPSC: spontaneous Excitatory PostSynaptic Current
TRPA1: Transient Receptor Potential A1 channel
TTX: tetrodotoxin
