# Supplementary figures and images for: TRPA1 channels promote astrocytic Ca2+ hyperactivity and synaptic dysfunction mediated by oligomeric forms of amyloid-β peptide
Source: Mol Neurodegener. 2017 Jul 6;12:53. doi: 10.1186/s13024-017-0194-8 (PMC5501536; doi:10.1186/s13024-017-0194-8)

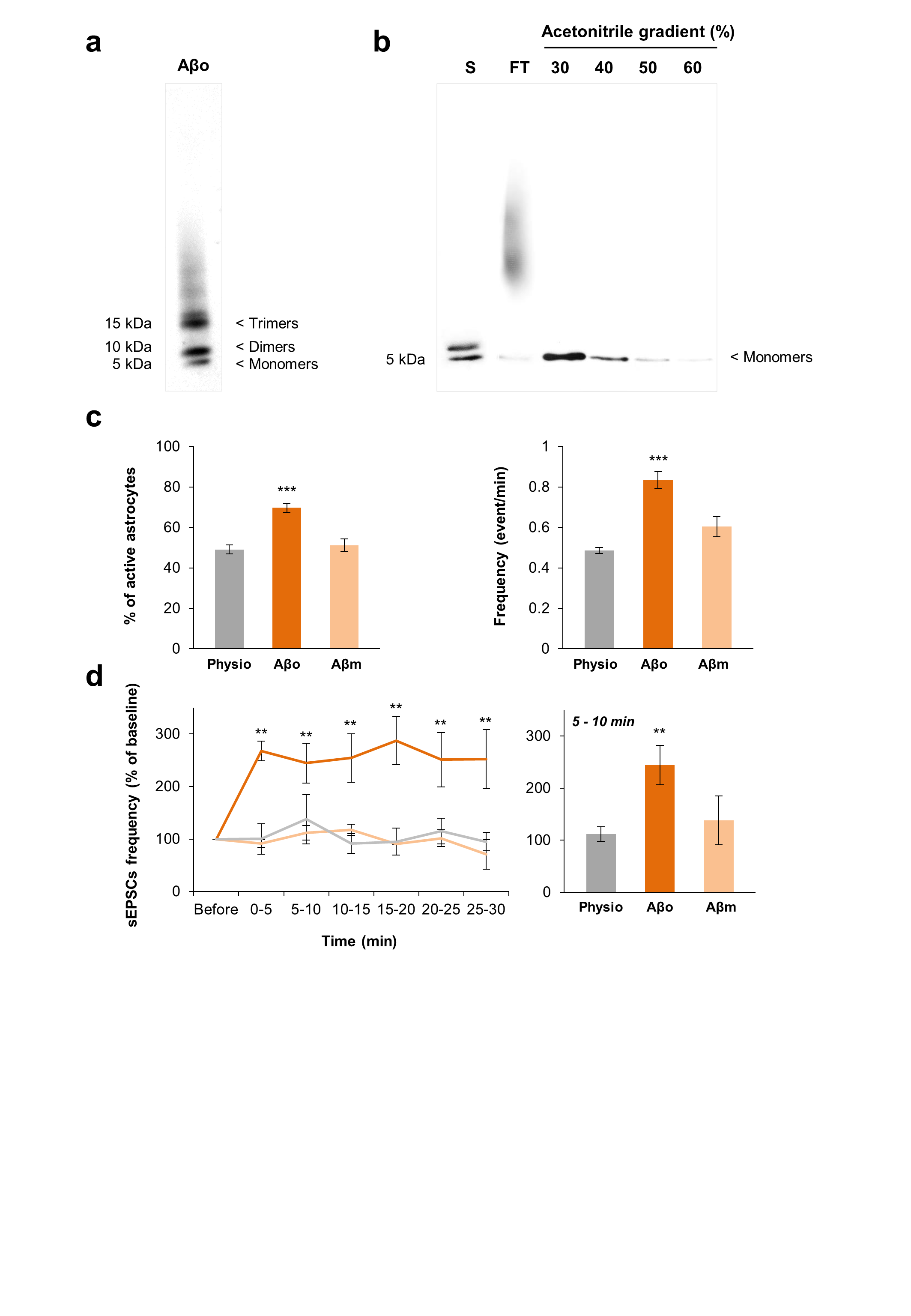

Supplement: Supplementary file 3 — Characterization and effects of Aβ oligomers and purified monomers. (a) SDS-PAGE analysis of Aβ oligomers examined by western-blot with 4G8 antibody. Aβo showed faintly monomers and abundant dimers and trimers. (b) SDS-PAGE analysis of Aβ monomers after purification on C18 column. All fractions were electrophoresed on 15% tris-glycine gel. Aβ monomer is mainly eluted at 30% acetonitrile. S, sample loaded; FT, flow through; peptides eluted at 30, 40, 50 and 60% acetonitrile. (c) Within the astrocytic population, proportion of astrocytes displaying calcium activity and frequency of astrocyte calcium activity in physiological condition (grey; n = 43), under 100 nM Aβo application (orange; n = 12) and under 100 nM Aβm application (light orange; n = 7). (d) Time course and histogram at 5–10 min of the frequency of sEPSCs in physiological condition (grey; n = 5), under application of 100 nM Aβo (orange; n = 7) or 100 nM Aβm (light orange; n = 5). In any case, Aβm was obtained from the 30% acetonitrile fraction purified in b. (TIFF 291 kb) [file 13024_2017_194_MOESM1_ESM.tif]

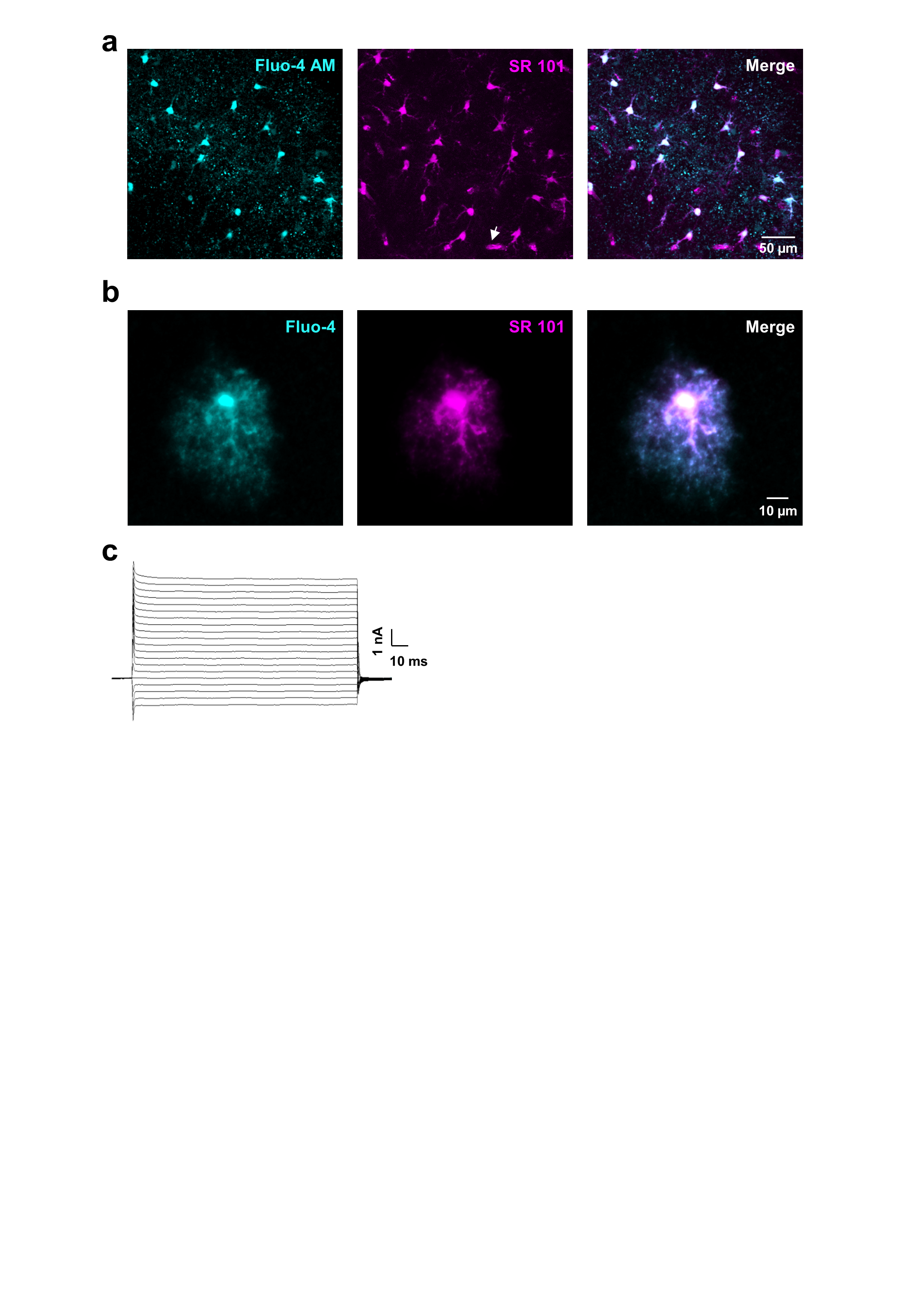

Supplement: Supplementary file 4 — Characterization of Fluo-4-loaded cells in the stratum radiatum of mouse coronal slice. (a) Confocal image of Fluo-4-loaded (cyan) and SR101-labeled (magenta) cells in the CA1 stratum radiatum. Merged image showing the proportion of loaded astrocytes (white), confirming that most of the loaded cells are astrocytes. One hour before slicing, animals were iv injected with SR101 as described previously [51]. Vessels are only labeled with SR101 (white arrow). (b) Z-stack projections of confocal images of a patched astrocyte loaded with Fluo-4 (cyan) and SR101 (magenta). Merged image showing the Fluo-4 diffusion in the whole astrocytic territory. (c) Example of a passive whole-cell current recorded in a stratum radiatum astrocyte. Cell was held at −70 mV and 10 mV hyper- and depolarizing voltage steps of 80 ms duration were applied (−110 to +80 mV). (TIFF 1907 kb) [file 13024_2017_194_MOESM2_ESM.tif]

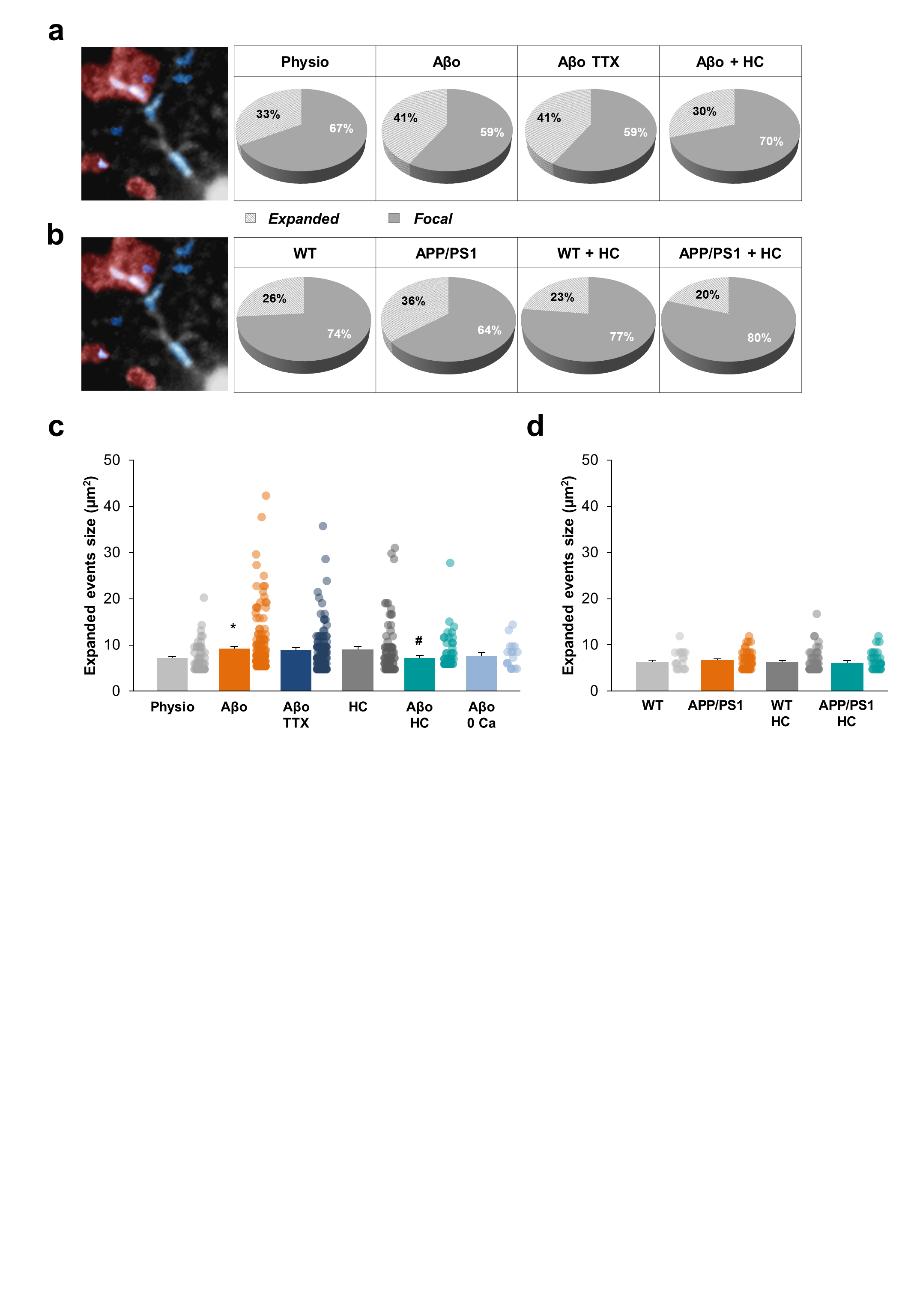

Supplement: Supplementary file 5 — Spatial properties of compartmentalized calcium events in the astrocytic processes. (a) Proportion of expanded/focal events in physiological condition, under 100 nM Aβo application, 100 nM Aβo + 500 nM TTX co-application and 100 nM Aβo + 40 μM HC 030031 co-application. (b) Proportion of expanded/focal events in APP/PS1–21 mice and their littermates (WT) in physiological condition or under 40 μM HC 030031 treatment. (c) Mean size of expanded Ca2+ events in physiological condition (grey), under 100 nM Aβo application (orange), 100 nM Aβo + 500 nM TTX co-application (dark blue), 40 μM HC 030031 (dark grey), 100 nM Aβo in Ca2+-free medium application (0 Ca; light blue) and 100 nM Aβo + 40 μM HC 030031 co-application (cyan). (d) Mean size of expanded Ca2+ events in APP/PS1–21 mice (orange) and their littermates (WT, light grey) in physiological condition or with 40 μM HC 030031 treatment (WT, dark grey and APP/PS1–21, cyan). Results are compared with the physiological condition with *, p < 0.05; **, p < 0.01 and ***, p < 0.001 or the Aβo condition with #, p < 0.05; ##, p < 0.01 and ###, p < 0.001. (TIFF 752 kb) [file 13024_2017_194_MOESM4_ESM.tif]

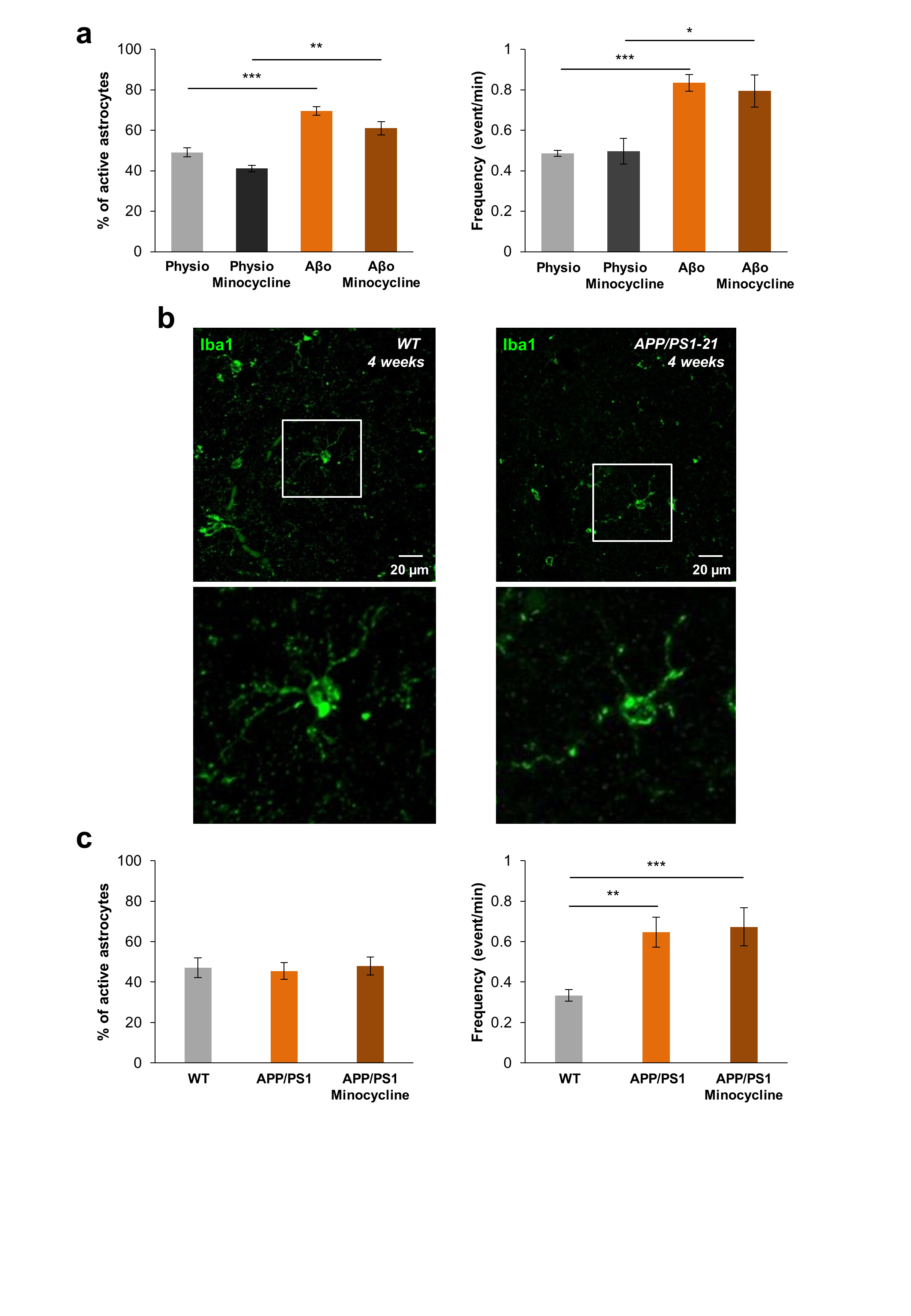

Supplement: Supplementary file 6 — Blockade of microglia activation with minocycline does not prevent the effect of Aβo on astrocyte Ca2+ activity. (a) Within the astrocytic population, proportion of astrocytes displaying calcium activity and frequency of astrocyte calcium activity in physiological condition (grey; n = 43), under 50 nM minocycline (dark grey; n = 5); 100 nM Aβo application (orange; n = 12) and under 50 nM minocycline +100 nM Aβo application (dark orange; n = 6). (b) Immunohistochemistry of mouse stratum radiatum microglia showing that Iba1-positive cells were not hypertrophic in one-month-old APP/PS1–21 mice when compared to WT littermates. Higher magnification of representative microglia is shown in the lower panels. (c) Proportion of astrocytes displaying calcium activity and frequency of astrocyte calcium activity in APP/PS1–21 mice (orange; n = 8), under 50 nM minocycline application (dark orange; n = 6) or in WT littermates (grey; n = 8). Minocycline is pre-incubated 15 min before recording. Results are compared with the physiological condition, with or without minocycline, or with the WT littermates with *, p < 0.05; **, p < 0.01 and ***, p < 0.001. (TIFF 1680 kb) [file 13024_2017_194_MOESM5_ESM.tif]

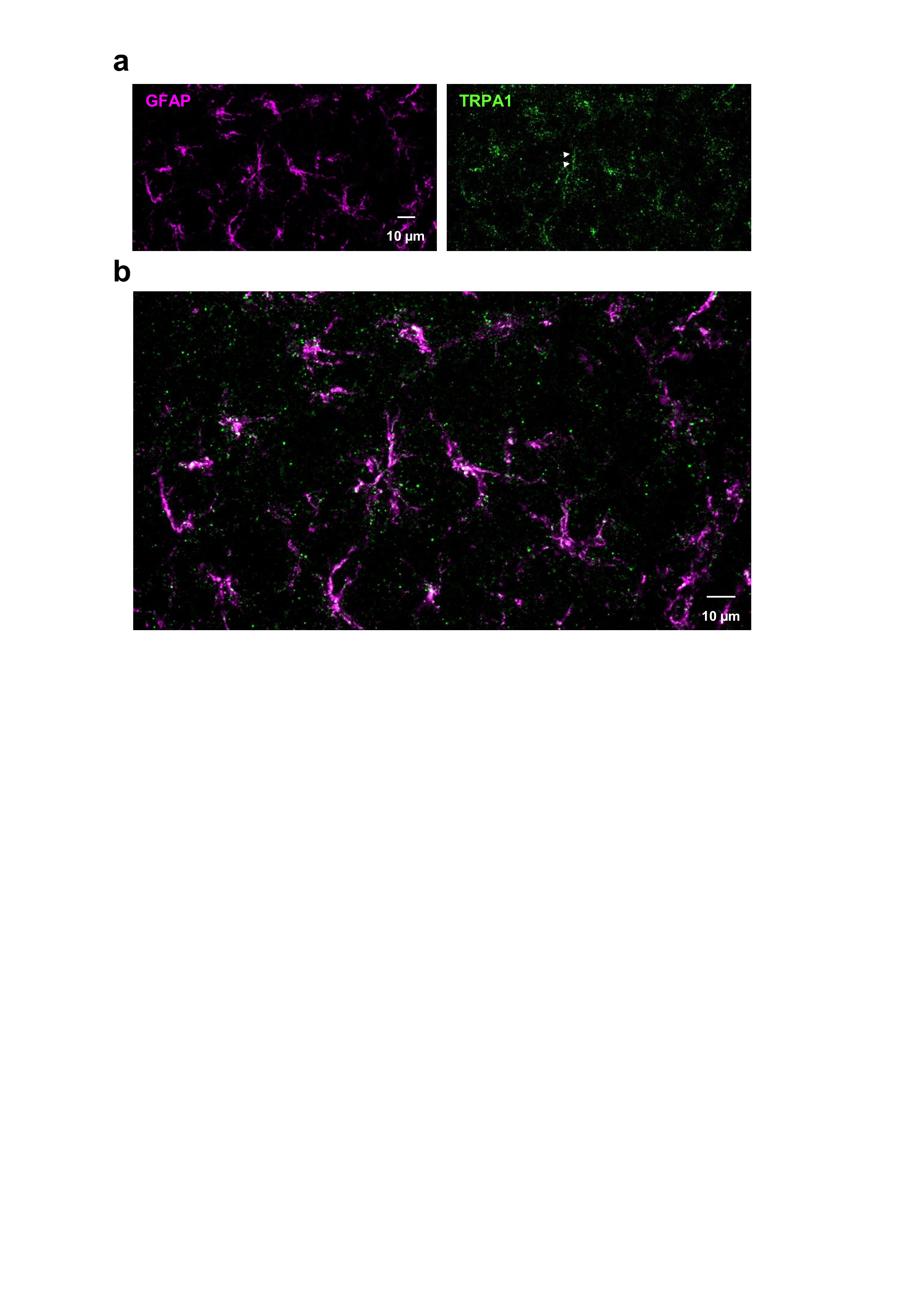

Supplement: Supplementary file 7 — TRPA1 is expressed in stratum radiatum astrocyte cell body and processes. (a) Immunohistochemistry of mouse stratum radiatum astrocytes showing that TRPA1 channels (green) are expressed within astrocytic domains certified by GFAP staining (magenta). (b) Merge image showing that most TRPA1 staining co-localized and surrounded GFAP-positive processes. (TIFF 2786 kb) [file 13024_2017_194_MOESM6_ESM.tif]
